# Reversible Phase Transformations in a Double-Walled Diamondoid Coordination Network with a Stepped Isotherm for Methane

**DOI:** 10.1021/jacs.4c03555

**Published:** 2024-06-21

**Authors:** Xia Li, Debobroto Sensharma, Leigh Loots, Shubo Geng, Sousa Javan Nikkhah, En Lin, Volodymyr Bon, Wansheng Liu, Zhifang Wang, Tao He, Soumya Mukherjee, Matthias Vandichel, Stefan Kaskel, Leonard J. Barbour, Zhenjie Zhang, Michael J. Zaworotko

**Affiliations:** † Department of Chemical Science, Bernal Institute, University of Limerick, Limerick V94 T9PX, Republic of Ireland; ‡ Department of Chemistry and Polymer Science, Stellenbosch University, Matieland 7602, South Africa; § College of Chemistry, Nankai University, Tianjin 300071, People’s Republic of China; ∥ Faculty of Chemistry, Technische Universität Dresden, Bergstrasse 66, 01062 Dresden, Germany

## Abstract

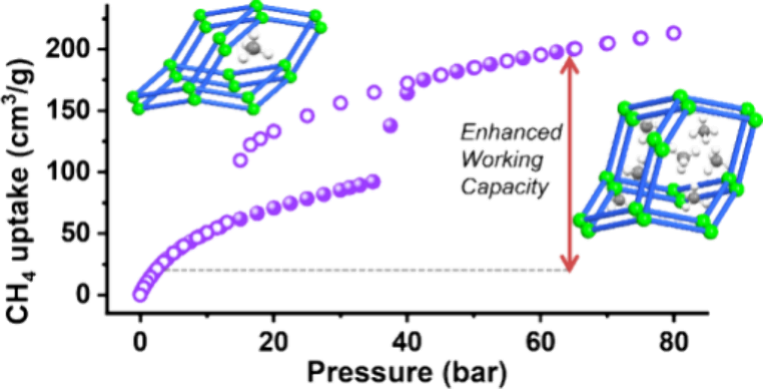

Flexible metal–organic materials (FMOMs) with stepped isotherms can offer enhanced working capacity in storage applications such as adsorbed natural gas (ANG) storage. Unfortunately, whereas >1000 FMOMs are known, only a handful exhibit methane uptake of >150 cm^3^/cm^3^ at 65 atm and 298 K, conditions relevant to ANG. Here, we report a double-walled 2-fold interpenetrated diamondoid (**dia**) network, **X-dia-6-Ni,** [Ni_2_L_4_(μ-H_2_O)]_*n*_, comprising a new azo linker ligand, **L**^**–**^ (**L**^**–**^ = (*E*)-3-(pyridin-4-yldiazenyl)benzoate) and 8-connected dinuclear molecular building blocks. **X-dia-6-Ni** exhibited gas (CO_2_, N_2_, CH_4_) and liquid (C8 hydrocarbons)-induced reversible transformations between its activated narrow-pore **β** phase and **γ**, a large-pore phase with *ca*. 33% increase in unit cell volume. Single-crystal X-ray diffraction (SCXRD) studies of the as-synthesized phase **α**, **β**, and **γ** revealed that structural transformations were enabled by twisting of the azo moiety and/or deformation of the MBB. Further insight into these transformations was gained from variable temperature powder XRD and *in situ* variable pressure powder XRD. Low-temperature N_2_ and CO_2_ sorption revealed stepped Type F–II isotherms with saturation uptakes of 422 and 401 cm^3^/g, respectively. **X-dia-6-Ni** exhibited uptake of 200 cm^3^/cm^3^ (65 atm, 298 K) and a high CH_4_ working capacity of 166 cm^3^/cm^3^ (5–65 bar, 298 K, 33 cycles), the third highest value yet reported for an FMOM and the highest value for an FMOM with a Type F–II isotherm.

## Introduction

**Scheme 1:**
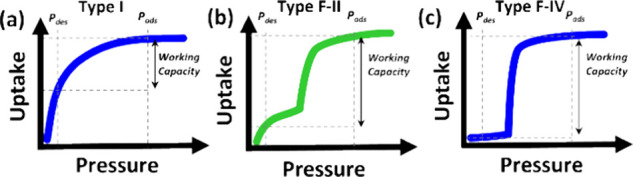
Examples of Adsorption Isotherms of Physisorbents: (a) Type I = Rigid microporous; (b) Type F–II = Flexible Microporous (Narrow to Large Pore); (c) Type F–IV = Flexible Microporous (Non-porous to Porous)

Metal–organic materials (MOMs) such as metal–organic frameworks (MOFs) and porous coordination polymers (PCPs) that are amenable to fine-tuning by crystal engineering are of interest as they can offer superior performance for gas/vapor/liquid storage and/or separation.^1−6^ There are now >120,000 MOF entries in the Cambridge Structural Database (CSD)^7^ with *ca*. 1000 known to be flexible metal–organic materials (FMOMs) as characterized by stimuli-induced structural transformations between low- and high-porosity phases.^8−11^ Whereas rigid microporous materials typically display Type I (Langmuir) adsorption isotherms,^12^ including for several high uptake methane sorbents,^13−18^ FMOMs can undergo structural phase transformations in response to external stimuli, such as exposure to gases or vapors.^19,20^ This in turn results in stepped sorption isotherms with a characteristic threshold “gate-opening” pressure (*P*_GO_, Scheme 1).^21−25^ Thanks to this characteristic, FMOMs have emerged as a class of porous materials with potential utility, especially for methane (adsorbed natural gas, ANG) storage.^21,26,27^ This potential is enhanced by the effects of flexibility, including improved heat management and higher working capacity (Type F–II and F–IV isotherms^21^ can offer higher uptake differences between the loading and release pressures than Type I isotherms, Scheme 1).^27^

Several design principles for FMOMs have emerged^11,28,29^ and phase transformation mechanisms have been classified into three categories: ligand contortion, e.g., bending, twisting, and rotation; molecular building block (MBB) coordination environment change, e.g., deformation and reconstitution/isomerism; relative motions of subnets, e.g., interpenetrated and layered net sliding or expansion.^10,11^ Nevertheless, several aspects of FMOMs remain understudied, including the relationships between ligand flexibility, topology, *P*_GO_, and uptake. Therefore, there is a need to explore new topologies and ligands for use in FMOMs.

With respect to topologies, **sql**, **dia**, **nbo**, and **kgm** topology networks are known to exhibit flexibility.^11^ One of the first studied and most accessible classes of MOMs, **dia** (diamondoid) coordination networks,^30^ can be built from 4-connected nodes and often exhibit interpenetration,^31^ a phenomenon that necessarily decreases the pore volume of porous MOMs.^32−34^ Around 30 flexible **dia** networks have been reported, including **SHF-61**,^35^**X-dia-1-Ni**,^21^**X-dia-1-Ni**_**0.89**_**Co**_0.11_,^36^**JUK-8**,^33^ and **X-dia-2-Cd.**(31) Several of these **dia** FMOMs are high performing with respect to ANG, including those of general formula ML_2_ where M = divalent metal cation, L = a linker ligand comprising an N-donor and a carboxylate moiety (N/CO_2_ linker).

In this contribution, we introduce a new N/CO_2_ linker ligand that comprises an azo moiety, (*E*)-3-(pyridin-4-yldiazenyl) benzoic acid (**HL**), which was synthesized by the Wallach reaction.^37^ Motivated by previous studies on the flexibility of azo moieties,^38^ our objective was to prepare flexible coordination networks based upon **L**^**–**^ and to determine if reversible structural transformations can be induced by exposure to various gases, vapors, and liquids. As reported herein, the outcome of the reaction of **HL** with NiCl_2_·6H_2_O was not the anticipated **dia** network of formula ML_2_ (M = divalent metal ion; L = linker). Rather, it was [Ni_2_L_4_(μ-H_2_O)]_*n*_, **X-dia-6-Ni**, a rare example^39−41^ of a double-walled **dia** network built from an 8-connected Ni dimer and spiro connections. The structural transformations and sorption properties of **X-dia-6-Ni** characterized by single crystal X-ray diffraction (SCXRD), *in situ* powder X-ray diffraction (PXRD), and gas sorption studies are reported herein.

## Results and Discussion

### Synthesis and Structural Analysis

**Figure 1:**
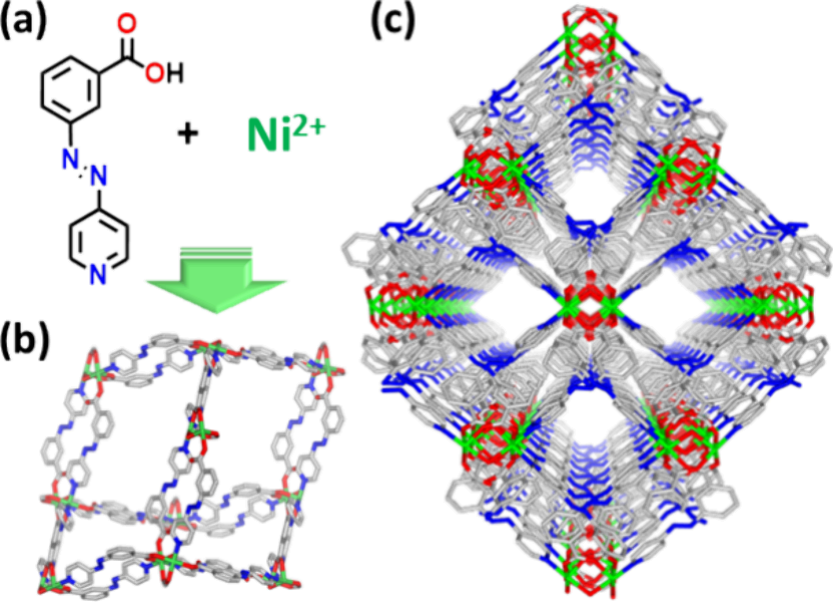
(a) Structure of the ligand (*E*)-3-(pyridin-4-yldiazenyl) benzoic acid (**HL**). (b) Double-walled adamantanoid cage in **X-dia-6-Ni**. (c) 1D quadrangular channels along the *b*-axis in the as-synthesized phase, **X-dia-6-Ni-α**.

**Figure 2:**
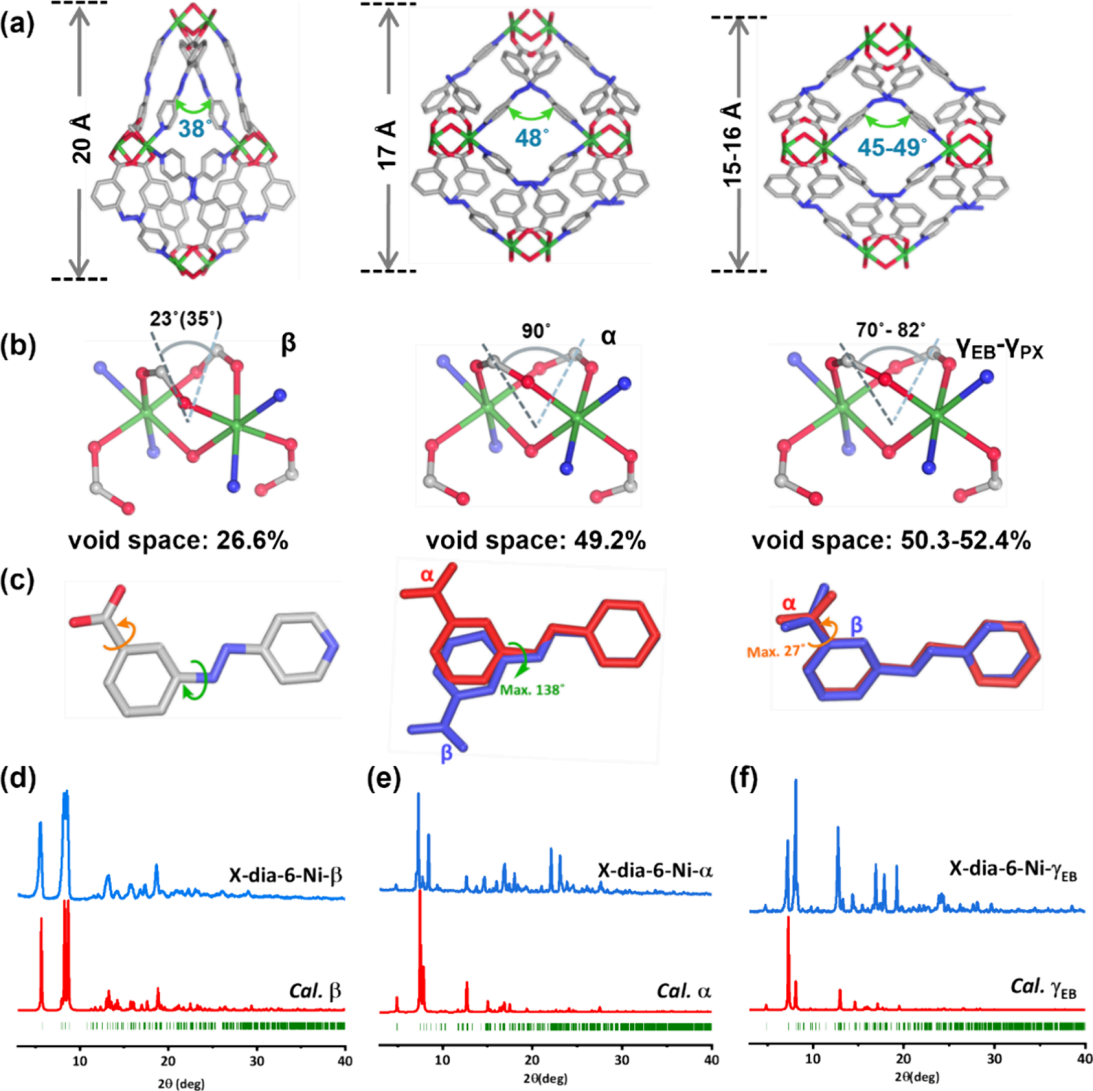
(a) Square portion of the **dia** nets in the low porosity (**β**) and high porosity (**α**) and higher porosity (**γ**_**EB**_-**γ**_**PX**_) phases of **X-dia-6-Ni**. (b) Deformations of MBBs in three phases. (c) Superposed representations of conformations **L**^**–**^ in **X-dia-6-Ni-α** (red) and **X-dia-6-Ni-β** (blue). Comparison of experimental PXRD patterns of (d) **β**, (e) **α**, and (f) **γ**_**EB**_ and PXRD patterns calculated from the SCXRD-determined structures.

The novel bifunctional linker ligand (*E*)-3-(pyridin-4-yldiazenyl) benzoic acid (**HL**) was synthesized by a facile procedure (see Supporting Information and Figure S1). Solvothermal reaction of **HL** and NiCl_2_·6H_2_O in dimethylformamide (DMF) and methanol (MeOH) at 60 °C afforded block-shaped orange-red crystals of the double-walled diamondoid (**dia**) network **X-dia-6-Ni-α** (Figure 1 and Figure S2). An SCXRD study of the as-synthesized crystals revealed that **X-dia-6-Ni-α** had crystallized in the orthorhombic space group *Fddd* with *a* = 27.6693(7) Å, *b* = 29.8486(6), *c* = 38.2589(8) Å, α = β = γ = 90°, *V* = 31597.6(12) Å^3^ (Table S1). The octahedral coordination environment of each Ni^2+^ center comprises four carboxylate O-donor atoms, three of them from **L**^**–**^ ligands (O1–O3), and one from a water molecule (O4) as well as two N-donor atoms (N1, N2) from two distinct **L**^**–**^ ligands (Figure S3). Each binuclear {Ni_2_} MBB^42^ comprises two Ni^2+^ cations bridged by a water molecule (μ_2_–OH_2_), two bidentate (η^2^) carboxylate groups, one monodentate (η^1^) carboxylate group, and two nitrogen atoms from two distinct **L**^**–**^ ligands, resulting in pseudo-octahedral geometry (Figure S3a). The {Ni_2_} MBB has the formula [Ni_2_(H_2_O)L_4_]_*n*_ and, although bonded to eight ligands, is 4-connected to adjacent MBBs (Figure S3b) because of spiro or “double-walled” connections. The prototype of this double-walled **dia** coordination network based upon the {Ni_2_} MBB, [Ni_2_(nicotinate)_4_(μ-H_2_O)] was reported by Lin’s group in 2001.^43^ Further, our review of the literature revealed 71 examples of such double walled structure in MOMs with **dia** topology (Table S2), only one of which has been reported to exhibit flexibility, in this case induced by solvent.^44^ In terms of the MBB, CSD database mining (version 5.44, April 2023) revealed 117 entries, of which 8 entries are 2D networks and 24 entries are 3D networks (Table S3). Five of these 3D networks exhibit **ddi** or “double diamondoid” topology, which should not be confused with the double-walled **dia** topology herein. Five of the 3D networks are doubled-walled **dia**, most of the remainder having some combination of double-walled and single-walled connections between MBBs (Table S3). To our knowledge, the only previous studies that investigated the flexibility of {Ni_2_} sustained coordination networks was our recently reported study on **ddi** topology networks.^45^**X-dia-6-Ni-α** exhibits 2-fold interpenetration with channels along the *b* axis with effective pore dimensions of ca. 17 Å × 11 Å and a calculated guest-accessible void volume of 49.2% (PLATON,^46^Figure 1b, Figure S4). The experimental PXRD pattern of a bulk sample of **X-dia-6-Ni-α** is consistent with that calculated from the SCXRD data (Figure 2e). Given the flexibility observed in single-walled **dia** and **ddi** topology networks, we were motivated to study if **X-dia-6-Ni-α** might also exhibit stimulus-induced phase transformations.

#### Activation

We observed that exchange with methanol and subsequent evacuation induced **X-dia-6-Ni-α** to undergo a structural transformation to a narrow pore phase, **X-dia-6-Ni-β**, as determined by SCXRD and PXRD (Figure 2). PXRD of the bulk **β** showed peak shifts in characteristic peaks (e.g., 2θ = 5.7° in **β**, 2θ = 4.9° in **α**, Figure 2d,e). SCXRD data revealed that **β** is a contorted version of the **α** phase, with the same connectivity and different unit cell parameters. Unlike **α** (*Fddd*), **X-dia-6-Ni-β** adopted space group *Fdd2* with 24.0% shrinkage of the unit cell volume (31597 to 24021 Å^3^, Table S1). The **α** to **β** transformation was accompanied by constriction of the quadrangular channels along the *b* axis with the ligand **L**^**–**^, channels being defined by edges drawn between O atoms from water molecules and Ni centers (Figure 2a). Whereas a negligible change was observed in the quadrilateral edge length (Δ*d*_*max*_ = 0.13 Å), the ∠Ni–O–Ni angle decreased from 48.38(1)° (**α**) to 37.66(2)° (**β**) (Figure 2a and Table S4). Concomitantly, one diagonal of the quadrilateral reduced from 10.660(1) Å (**α**) to 8.480(3) Å (**β**), whereas the other expanded from 17.073(6) Å to 18.67 Å (**α**) to 20.317(13) Å (**β**) (Table S4). The transformation from **α** to **β** can be attributed to deformations in both MBB and **L**^**–**^. The Ni^2+^ centers in **X-dia-6-Ni-β** retained octahedral coordination geometries but an obvious “butterfly-like” flapping deformation occurred in the coordination environment of the MBB. The dihedral angles of two planes formed by two oxygens and one carbon atom in each bidentate carboxyl group within the same MBB decreased drastically from 89.61(46)° to 22.55(18)° (34.69(17)°) (**α** to **β**, Figure 2b and Table S4).

Conformational variation in **L**^**–**^ was another source of flexibility in **X-dia-6-Ni**. A “hinge-like” twist occurred in the azo moiety in the **α** to **β** transformation process, the azo bond acting as an axle with the dihedral angle of planes formed by the benzoate ring and pyridine ring reducing from 43.33(56)°; 17.71(26)° to 50.59(71)°, (40.63(40)°); 23.90(40)°, (7.67(47)°). The maximum twist angle of **L**^**–**^ was found to be ca. 138° (Figure 2c and Table S4, with multiple values coming from lower symmetry of **β**). In addition, the C(benzoate)-C(carboxyl) bond connecting the carboxyl group and benzoate ring in **L**^–^ twisted during transformation from **α** to **β**, the maximum dihedral angle of the carboxyl plane and benzoate plane between these two phases being ca. 27° (Figure 2c). In effect, the “butterfly-like” flapping motion of the MBB and twisting around the azo and carboxyl moieties in **L**^–^ worked synergistically to compress the rectangular channel, resulting in a reduction of the guest-accessible volume from 49.2% (**α**) to 26.6% (**β**) (Figure S4).

The bulk purity of **X-dia-6-Ni-β** accompanying the structural transformation was monitored by PXRD (Figure 2e). Variable temperature PXRD (VT-PXRD) conducted on **X-dia-6-Ni-α** under nitrogen flow revealed a phase change from **α** to **β** at 373 K (Figure S5). The structural transformation between **α** and **β** associated with guest release was found to be reversible, with **β** reverting to **α** after soaking in DMF/MeOH at 60 °C for 1 day (Figure S6).

#### C8 Hydrocarbon and Solvent Loading

After gaining structural insight into the mechanism of the unit cell volume shrinkage of **X-dia-6-Ni** during transformation from **α** to **β**, we explored the flexibility in **X-dia-6-Ni**. **X-dia-6-Ni** by exposure to guests which possess larger molecular volumes than the solvent used for synthesis, DMF and MeOH (85.1 and 40.9 Å^3^). *para*-Xylene (PX, 133.2 Å^3^), *meta*-xylene (MX, 127.6 Å^3^), *ortho*-xylene (OX, 124.1 Å^3^), and ethylbenzene (EB, 130.6 Å^3^) each resulted in unit cell volume expansion (solvent molecular volumes were calculated by XSeed^47^). SCXRD data revealed four crystal structures with cell volumes larger than those of **X-dia-6-Ni-α**: **X-dia-6-Ni-γ**_**EB**_, **X-dia-6-Ni-γ**_**MX**_, **X-dia-6-Ni-γ**_**OX**_, and **X-dia-6-Ni-γ**_**PX**_.

Previous studies of FMOMs have revealed only a few examples in which bulkier guests like C8 hydrocarbons resulted in phases with higher porosity than the corresponding as-synthesized phases.^25,48−51^ The **γ**_**EB**_, **γ**_**MX**_, **γ**_**OX**_, and **γ**_**PX**_ phases herein are also 2-fold interpenetrated **dia** networks with unit cell volumes of 32,399, 32,471, 32,618, and 33,546 Å^3^ for **γ**_**EB**_–**γ**_**PX**_, respectively (Table S1). This compares with 31,598 Å^3^ for **α**. The *b* axis changed from 29.85 Å in **α** to 33.02 Å, 32.47, 32.93, and 34.59 Å in **γ**_**EB**_–**γ**_**PX**_, respectively (Table S1). The corresponding solvent-accessible void volumes were determined to be 49.2%, 50.3%, 52.1%, 51.5%, and 52.4% for **α** and **γ**_**EB**_–**γ**_**PX**_, respectively (Figure S4). Analysis of the SCXRD results revealed that both the MBB and **L**^**–**^ in **X-dia-6-Ni-γ** underwent conformational changes compared to those in **X-dia-6-Ni-α**. During the transformation from **α** to **γ**_**EB**_, the MBB in **γ**_**EB**_ underwent further “butterfly-like” deformation with the dihedral angles of two planes formed by two oxygens and one carbon in each bidentate coordinated carboxyl groups of one MBB changing from 89.61(46)° to 76.11(42)° (Table S4). Simultaneously, a twist occurred in **L**^**–**^ around the −N=N– bond and the dihedral angles formed by the benzoate plane and the pyridine plane of **L**^**–**^ changed from 43.33(56)°; 17.71(26)° in **α** to 33.03(19)°; 18.29(24)° in **γ**_**EB**_ (Table S4). Another twist in **L**^**–**^ around the C(benzoate)-C(carboxyl) bond was also found, with dihedral angles between the carboxyl plane and benzoate plane changing from 13.76(36)°, 3.10(42)° in **α** to 13.75(21)°, and 7.83(20)° in **γ**_**EB**_ (Table S4). Overall, the **X-dia-6-Ni-γ** phases exhibited both the twisting of ligands and the deformation of MBBs to enable framework expansion. The PXRD patterns of activated **X-dia-6-Ni** before and after soaking in C8 showed peak shifting (Figure 2e,f and Figure S7), the breathing effect monitored by PXRD being consistent with the SCXRD results. PX, MX, and OX sorption isotherms were collected at 298 K from *P*/*P*_0_ = 0–95% relative pressure and exhibited stepped isotherms consistent with phase transformations (Figure S8). Structural transformation can also be induced by solvent, as indicated by PXRD patterns of tetrahydrofuran (THF) and ethanol (EtOH) soaked samples (Figure S9).

#### Thermal Stability and Fourier Transform Infrared (FTIR) analysis

The bulk phase purities of **α**, **β**, and **γ** phases were confirmed by matching experimental and calculated PXRD patterns (Figure 2d–f, Figure S7). The thermal stability of **X-dia-6-Ni-α** was evaluated by thermogravimetric analysis (TGA) and VT-PXRD. TGA results revealed that **X-dia-6-Ni-α** exhibited a mass loss of 34.4% at 175 °C with no further weight loss until 360 °C (Figure S10). TGA conducted on **X-dia-6-Ni-β** showed a negligible mass loss. TGA results for **γ**_**EB**_, **γ**_**MX**_, **γ**_**OX**_, and **γ**_**PX**_ were similar to those of **α**, with mass losses of 26.6–32.8% at 181–196 °C (Figure S10). VT-PXRD conducted on **X-dia-6-Ni-α** under N_2_ flow indicated that phase transformation to **β** had occurred at 373 K and that **β** was stable to 433 K (Figure S5). Further, VT-PXRD conducted on **X-dia-6-Ni-β** from 298 to 433 K indicated no phase transformations upon temperature ramping (Figure S11). FTIR studies indicated guest release from **α** to **β** with the disappearance of characteristic C=O stretching peaks of DMF at 1663 cm^–1^ in **β** (Figure S12). The appearance of aromatic ring C–H stretching peaks of xylenes at 3070 to 2865 cm^–1^ in the FT-IR spectra of **γ**_**EB**_, **γ**_**MX**_, **γ**_**OX**_, and **γ**_**PX**_ is consistent with loading of C8 guests in **γ** phases.

#### Gas Sorption

**Figure 3:**
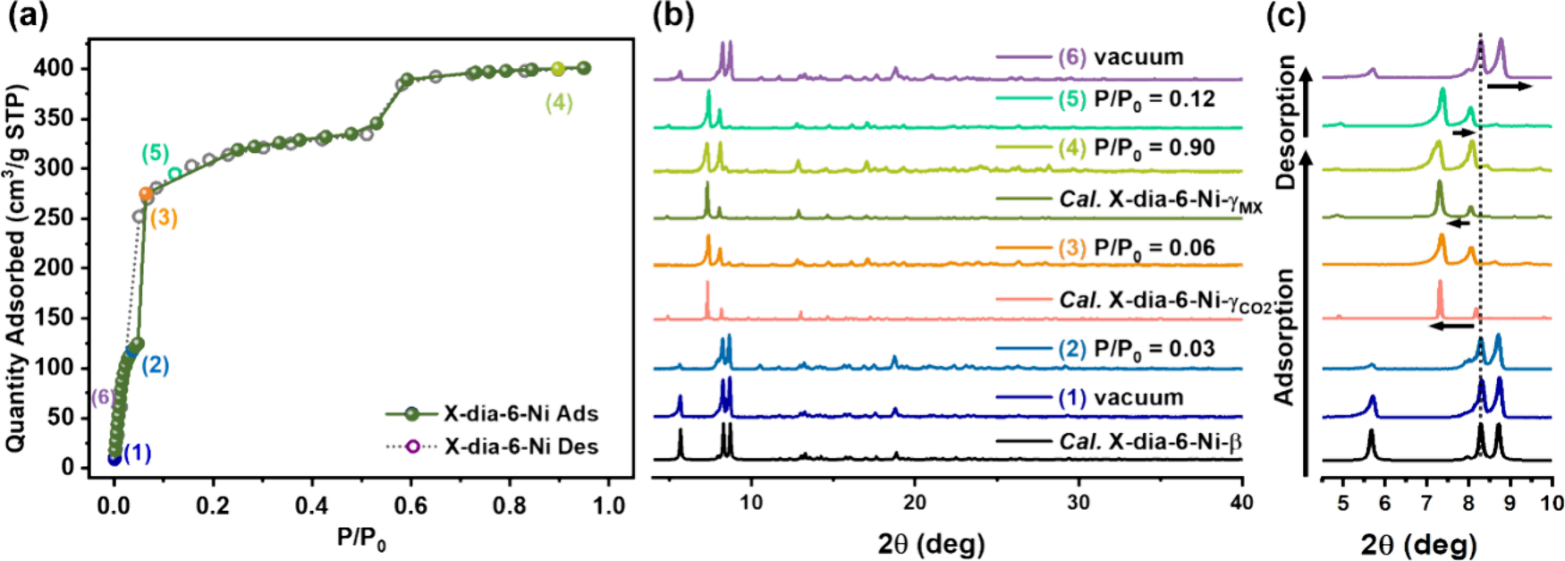
(a) CO_2_ sorption isotherm of **X-dia-6-Ni** at 195 K from *P*/*P*_0_ = 0–1. (b) Comparison of experimental *in situ* variable pressure PXRD patterns of **X-dia-6-Ni** collected at different CO_2_ loading pressures, 195 K and PXRD patterns calculated from single crystal structures of **X-dia-6-Ni-β**, **X-dia-6-Ni-γ**_**MX**_, and simulated structure of **X-dia-6-Ni-γ**_**CO2**_, respectively. (c) PXRD patterns from 4 to 10° 2θ for **X-dia-6-Ni**.

Since the transformations from **α** to **β** and **α** to **γ** can be induced by evacuation and solvent inclusion, we anticipated that gas molecules might also trigger phase transformations in **X-dia-6-Ni**. Low temperature N_2_ (77 K) and CO_2_ (195 K) isotherms both revealed steps and similar uptake. The CO_2_ data provided access to the intermediate phase over a broader pressure range, which we attribute to its larger quadrupole moment (13.4 × 10^–40^ cm^2^) and small kinetic diameter (3.3 Å).^21^ CO_2_ was therefore chosen to probe the phase transformations of **X-dia-6-Ni**. Sorption tests conducted on **X-dia-6-Ni-β** (activated at 333 K under dynamic vacuum) at 195 K displayed a 2-step isotherm with *P*_GO_ = 0.048 and 0.479 (*P*/*P*_0_), and a saturation CO_2_ uptake of 401 cm^3^/g (Figure 3a). The experimental pore volumes calculated from the CO_2_ isotherm (195 K), 0.196 and 0.600 cm^3^/g, which were obtained at *P*/*P*_0_ = 0.042 and 0.843, respectively, matched well with calculated pore volumes from SCXRD data of **β** (0.195 cm^3^/g) and **γ**_**MX**_ (0.608 cm^3^/g, Table S5). A CO_2_ sorption isotherm collected at 195 K on **X-dia-6-Ni-β** that had been activated at 353 K under a vacuum afforded a similar isotherm, indicating that the activation temperature **X-dia-6-Ni** does not necessarily affect structural flexibility (Figure S13). To further investigate these phase transformations, a high-pressure CO_2_ sorption experiment was conducted on **X-dia-6-Ni-β** from 0 to 35 bar at 298 K. A similar isotherm profile with gate-opening pressures at 3 and 29 bar was observed (Figure S14). **X-dia-6-Ni** reversibly transformed between its low and high-porosity phases with pressure varying from 2 to 10 bar for at least 3 cycles without significant uptake loss (Figure S15). The water sorption isotherm also revealed steps with *P*_GO_ at 39% relative humidity (RH) and 78% RH, but with a large hysteresis and no desorption at 0% RH. PXRD patterns collected after water sorption indicated an amorphous phase (Figure S16).

We next collected *in situ* PXRD and sorption coincident measurements on **X-dia-6-Ni-β** under CO_2_ from 0 to 1 bar at 195 K. The resulting data reveal that **X-dia-6-Ni** underwent a reversible structural transformation between its low and high porosity phases. During CO_2_ loading, the characteristic low angle 5.7° peak of **β** diminished as the 4.8° peak of **γ**_**MX**_ appeared at *P*/*P*_0_ = 0.90 (Figure 3b,c, Figure S17). The PXRD pattern of the intermediate CO_2_ loaded phase (*P*/*P*_0_ = 0.06), **X-dia-6-Ni-γ**_**CO2**_, was indexed and fitted using Le Bail refinement (SI Section 11, Figure S18). The refinement indicated that **γ**_**CO2**_ has a lower unit cell volume, 31,804 Å^3^, than **γ**_**MX**_, 32,471 Å^3^. The close match of PXRD patterns calculated from the single crystal structure of **γ**_**MX**_, the simulated CO_2_-loaded crystal structure and the *in situ* PXRD data collected at *P*/*P*_0_ = 0.9 indicates that they possess the same structure (Figure S19). The CO_2_ desorption process resulted in a phase transformation from **γ**_**CO2**_ to **β**. The reversibility of **X-dia-6-Ni** is illustrated by its transformation from its narrow pore **β** phase to the large pore **γ**_**MX**_ phase during CO_2_ adsorption and reversion to **β** during desorption. In addition, **X-dia-6-Ni** exhibited high saturation N_2_ uptake (422 cm^3^/g) at 77 K with gate-opening for N_2_ at *P*/*P*_0_ = 0.038 and an uptake of 170 cm^3^/g (Figure S20). The relatively high uptakes and recyclability suggested to us that **X-dia-6-Ni** could be a candidate for methane storage.

#### Methane Storage

As the main component of NG, methane is an alternative to petroleum as a transportation fuel and plays an important role in power generation.^2,13,52^ However, to serve as a transportation fuel, methane must be stored. Current approaches to methane storage, liquefied and compressed natural gas (LNG and CNG, respectively),^53,54^ are energy-intensive and have safety issues. ANG could offer a safer and less energy intensive approach to store methane.^55^ ANG performance can be assessed through the working capacity between the release (5 bar) and storage (35 or 65 bar) pressures.^56,57^ According to the US Department of Energy, the target of solid sorbents for ANG is 0.5 g/g or 350 cm^3^/cm^3^ at 298 K and 65 bar. These values are equivalent to the energy density of CNG at 250 bar.^58,59^

High surface area rigid sorbents have been targeted for ANG, which typically means Type-I sorption isotherms.^13−17^ Unfortunately, as discussed earlier (Scheme 1), a significant quantity of methane is retained at the release pressure of 5 bar.^56,60^ For instance, **HKUST-1** has been reported to exhibit CH_4_ uptake of 267 cm^3^/cm^3^ at 65 bar, but, due to its Type I isotherm, the working capacity was found to be only 190 cm^3^/cm^3^.^61−63^ Similarly, **MAF-38** was found to have uptake of 263 cm^3^/cm^3^ at 65 bar, but the working capacity was reported to be just 187 cm^3^/cm^3^.^64^ The number of FMOMs reported to possess a high methane working capacity remains limited. Indeed, to our knowledge, only 19 FMOMs^3,36^ exhibit structural transformation between low and high-porosity phases with appropriate *P*_GO_ values (Table S6). In particular, only six FMOMs with methane uptake >150 cm^3^/cm^3^ at 298 K have thus far been reported, five with Type F–IV isotherms: **MIL-53(Al)–OH** reported by Zhao’s group;^26^**Co(bdp)** and **Fe(bdp)** from Long’s group;^27^**X-dia-1-Ni**^21^ and **X-dia-1-Ni**_**0.89**_**Co**_**0.11**_(36) studied by our group (Table S6). In addition, the archetypal FMOM **MIL-53(Al)** exhibited a Type I isotherm up to 35 bar at 298 K with steps corresponding to phase transformations at temperatures below 213 K.^65,66^

**Figure 4:**
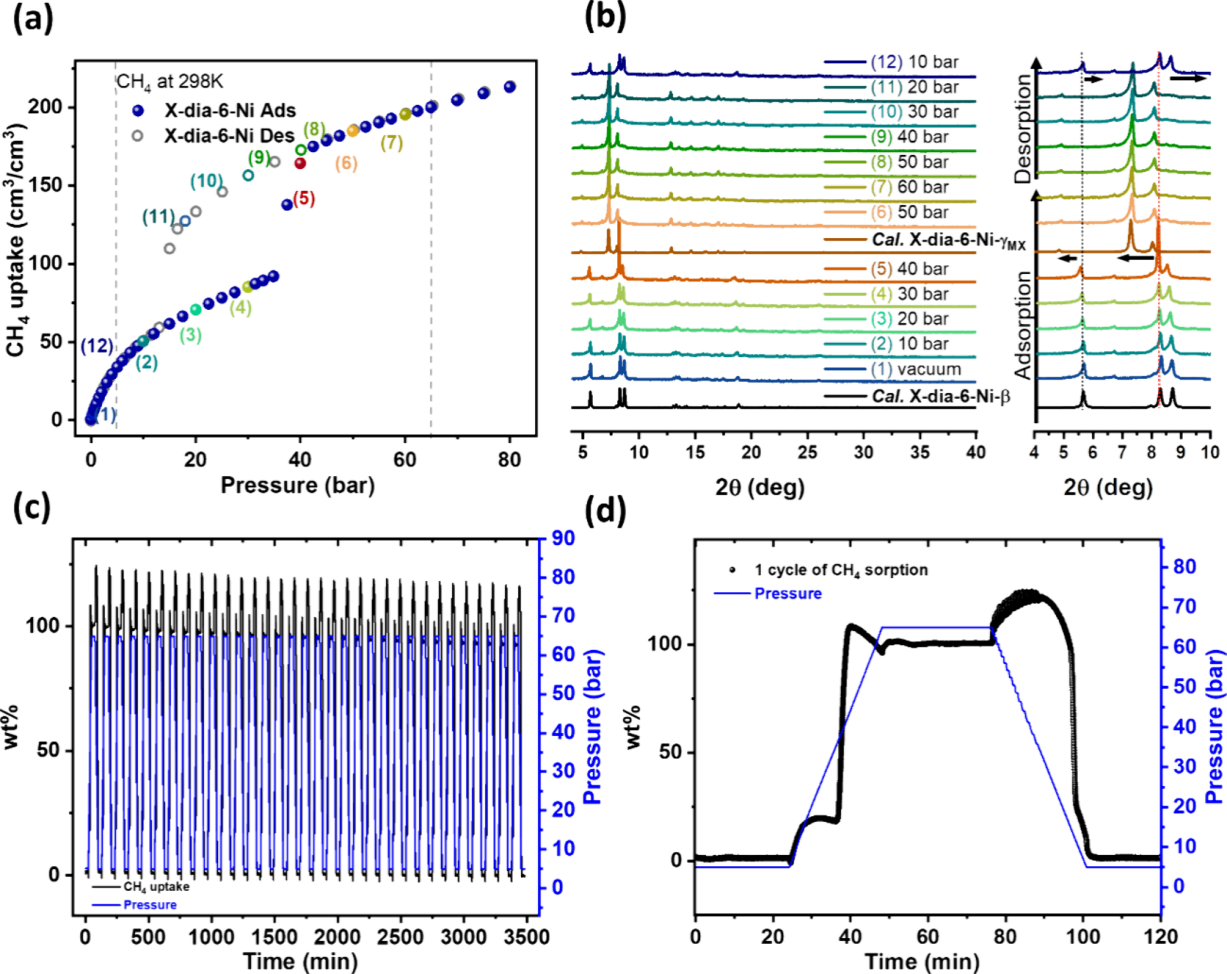
(a) Sorption isotherms for **X-dia-6-Ni** of CH_4_ 0–80 bar at 298 K. (b) Comparison of experimental *in situ* variable pressure PXRD patterns of **X-dia-6-Ni** collected at different CH_4_ loading pressures, 298 K and PXRD patterns calculated from single crystal structures of **X-dia-6-Ni-β**, **X-dia-6-Ni-γ**_**MX**_, and PXRD patterns from 4 to 10° 2θ for **X-dia-6-Ni**. (c) Dynamic adsorption–desorption CH_4_ sorption isotherms (black) of **X-dia-6-Ni** 33 cycles between 5 and 65 bar at 298 K. (d) One cycle of methane sorption from 5 to 65 bar at 298 K for **X-dia-6-Ni**.

To study the methane storage properties of **X-dia-6-Ni**, high-pressure methane sorption experiments were conducted at 298, 285, and 273 K from 0 to 80 bar (Figure 4a and Figure S21). The CH_4_ sorption isotherms measured at 298 K revealed a Type F–II isotherm with an inflection consistent with a narrow pore to large pore transformation and methane uptake of 213 cm^3^/cm^3^ (251 cm^3^/g) at 80 bar. Isotherms collected at 298, 285, and 273 K initially exhibited gradual but low CH_4_ uptake followed by abrupt increases. When the temperature of CH_4_ sorption was decreased from 298 to 285 and 273 K, *P*_GO_ was observed to shift to lower pressure, from ca. 35 to 30 and 27 bar, and the total uptake at 80 bar increased from 213 to 226 and 237 cm^3^/cm^3^, respectively (Figure S21). The CH_4_ uptake at 298 K from the desorption isotherm of **X-dia-6-Ni** was found to be 34 cm^3^/cm^3^ (40 cm^3^/g) at 5 bar, while that obtained from the adsorption isotherm at 65 bar was 200 cm^3^/cm^3^ (235 cm^3^/g), indicating a CH_4_ working capacity from 5 to 65 bar at 298 K of 166 cm^3^/cm^3^ (195 cm^3^/g). This working capacity ranks **X-dia-6-Ni** in the top three FMOMs in terms of volumetric methane working capacity, lower only than Co(bdp) (197 cm^3^/cm^3^) and Fe(bdp) (190 cm^3^/cm^3^).^27^

To obtain insight into the phase transformations of **X-dia-6-Ni** associated with high-pressure CH_4_ sorption, *in situ* variable-pressure PXRD studies were conducted. An activated sample **X-dia-6-Ni-β** was loaded into a capillary and then exposed to CH_4_ at pressures ranging from 0 to 60 bar at 298 K. Structural flexibility was suggested by shifting characteristic peak positions. The PXRD data indicated that, in the methane adsorption process, the low porosity **β** phase was maintained from 0 to 40 bar with its characteristic low angle peak observed at 5.7°. When CH_4_ pressure reached 50 bar, **β** transformed to the high porosity **γ**_**MX**_ phase, and the characteristic peak of **γ**_**MX**_ at 4.8° appearing as the peak of **β** at 5.7° disappeared (Figure 4b, Figure S22). During the CH_4_ desorption process from 60 to 20 bar **X-dia-6-Ni** remained as **γ**_**MX**_, but at 10 bar, **X-dia-6-Ni** reverted to **β**. The *in situ* PXRD results were therefore consistent with the methane-induced reversible transformation between **β** and **γ**_**MX**_. Analysis of the crystal structures collected at 0 bar and after CH_4_ loading indicates that the phase transformation mechanism can be attributed to ligand twisting around azo bonds and {Ni_2_} MBB deformations (Table S4).

The working capacity of **X-dia-6-Ni** was evaluated by cycling experiments between loading pressure (5 bar) and unloading pressure (65 bar) for >30 cycles with an uptake loss of 7.9% (Figure 4c). We attribute the decreased 5–65 bar working capacity to a shift of inflection pressure points resulting from smaller particle size (Figure S23) and partial loss of crystallinity during cycling (Figure S24). The cycling tests also showed that **X-dia-6-Ni** has relatively fast kinetics of methane adsorption and desorption between 5 and 65 bar at 298 K. Whereas both the adsorption and desorption processes of **X-dia-6-Ni** were rate limited by the pressure ramp rate of the sorption instrument, they had occurred within 24 min of the step onset in each case (Figure 4d). The PXRD pattern of **X-dia-6-Ni** before and after 33 cycles of methane loading and unloading suggested a partial loss of crystallinity (Figure S24).

To explore the transformation of **X-dia-6-Ni-β** to **X-dia-6-Ni-γ** during CH_4_ adsorption, we performed density functional theory (DFT) calculations, canonical Monte Carlo (CMC) and grand canonical Monte Carlo (GCMC) simulations (see Supporting Information for details). Experimentally, a partial transition from the **X-dia-6-Ni-β** to **X-dia-6-Ni-γ** happens between 30 and 40 bar during adsorption (Figure 4b), and from **X-dia-6-Ni-γ** to **X-dia-6-Ni-β** between 20 and 10 bar during CH_4_ desorption. To elucidate the maximum adsorption capacity of both phases, adsorption isotherms were predicted via GCMC simulations on 2 × 1 × 1 supercells of the DFT optimized primitive cells (see Figures S25 and S26). GCMC simulations reveal the maximum uptake is about 100 cm^3^/cm^3^ for **X-dia-6-Ni-β**, in good agreement with the experimental adsorption isotherm at 35 bar, whereafter, a framework transition to a more open phase is required to take up more CH_4_. Furthermore, **X-dia-6-Ni-γ** has a maximum uptake of about 300 cm^3^/cm^3^ at 35 bar (Figure S26). By correlating the simulated data with the experimental data, we can conclude that there is a phase transformation occurring around 35 bar. Interestingly, the empty open phase is about 1.11 eV per primitive unit cell higher in energy than the empty closed phase, corroborating the crucial role of adsorbate—host interactions and sufficient adsorbate pressure to open the framework. Nevertheless, subsequent adsorption/desorption runs show a loss of working capacity, crystallinity, and crystal size distribution (Figure 4 and Figure S24), which are indications of the presence of collapsed pores, and structural defects (uncoordinated metals and linkers). To validate these hypotheses for the primitive unit cell of **X-dia-6-Ni-γ**, one decoordinated ligand/MBB is introduced by breaking the Ni–N bond (i.e., Ni and the pyridine-nitrogen). Breaking up the Ni–N bond from 2.10 Å to over 3.05 Å is still less than 0.90 eV uphill on the potential energy surface. Partial decoordination or elongation of the Ni–N bond from 2.10 up to 2.67 Å is only endothermic by 0.60 eV. These results underscore the thermodynamic likelihood of such processes, which can result in partial loss of crystallinity, due to collapsed pores and structural defects. By lowering the temperature, decoordination can be inhibited, which also increases the working capacity (Figure S21).

## Conclusions

We report herein structural transformations between low-porosity and high-porosity phases of a guest-responsive coordination network, **X-dia-6-Ni**, with double-walled **dia** topology. The structures of the phases induced by gas and solvent exposure were structurally characterized using SCXRD and *in situ* PXRD studies, providing insight into the mechanism of flexibility of **X-dia-6-Ni**, which was driven by ligand twisting around azo bonds and {Ni_2_} MBB deformations. Most notably, a high deliverable capacity of methane, 166 cm^3^/cm^3^ (195 cm^3^/g) was achieved between 5 and 65 bar at 298 K with a Type F–II isotherm. This work suggests that Type F–II sorbents are worthy of further consideration for ANG even though they have measurable methane uptake in their narrow pore phases. Computational modeling confirms the high working capacity and suggests that performance could be improved by enabling a more facile transformation between narrow pore and large pore phases. With respect to crystal engineering, the combination of rotatable azo moieties in linker ligands and the deformable {Ni_2_} MBB mean that **X-dia-6-Ni** offers a platform to construct families of double-walled **dia** frameworks with high structural flexibility. Indeed, the amenability of both single- and double-walled **dia** nets to crystal engineering approaches through ligand or metal substitution will likely afford numerous families of flexible sorbents that exhibit stimulus-induced phase transformations. That this study, based upon exploiting the flexibility of azo linkers to enable narrow pore to large pore flexibility, resulted in competitive ANG storage performance suggests that such sorbents are worthy of further study.

## Supporting Information Available

Materials and methods, supporting figures, supporting tables, and supporting references (PDF)
X-ray data for **X-dia-6-Ni-α**; X-ray data for **X-dia-6-Ni-β**;X-ray data for **X-dia-6-Ni-γ**_**EB**_**;** X-ray data for **X-dia-6-Ni-γ**_**MX**_**;** X-ray data for **X-dia-6-Ni-γ**_**OX**_**;** X-ray data for **X-dia-6-Ni-γ**_**PX**_ (CIF)
**X-dia-6-Ni** simulated CO_2_ full loaded (CIF)
****X-dia-6-Ni-β**** with_Qeq_point_charges and **X-dia-6-Ni-γ**_**MX**_ with_Qeq_point_charges (CIF)

## Supplementary Material

ja4c03555_si_001.pdf

ja4c03555_si_002.cif

ja4c03555_si_003.cif

ja4c03555_si_004.cif
