# Supplementary material for: Reversible Phase Transformations in a Double-Walled Diamondoid Coordination Network with a Stepped Isotherm for Methane
Source: J Am Chem Soc. 2024 Jun 21;146(27):18387–95. doi: 10.1021/jacs.4c03555 (PMC11240251; doi:10.1021/jacs.4c03555)
Supplement: Supplementary file 1 — ja4c03555_si_001.pdf [file ja4c03555_si_001.pdf]

Supporting Information

Reversible Phase Transformations in a Double-Walled Diamondoid Coordination Network with a Stepped Isotherm for Methane

Xia Li,[a] Debobroto Sensharma,[a] Leigh Loots,[b] Shubo Geng,[c] Sousa Javan Nikkhah,[a] En Lin,[c] Volodymyr Bon,[d] Wansheng Liu,[c] Zhifang Wang,[c] Tao He,[a] Soumya Mukherjee,[a] Matthias Vandiche,[a] Stefan Kaskel,[d] Leonard J. Barbour,[b] Zhenjie Zhang,[c]* Michael J. Zaworotko[a]*

[a]Department of Chemical Science, Bernal Institute, University of Limerick, Limerick, V94 T9PX, Republic of Ireland
[b]Department of Chemistry and Polymer Science, Stellenbosch University, Matieland 7602, South Africa
[c]College of Chemistry, Nankai University, Tianjin 300071, People's Republic of China
[d]Faculty of Chemistry, Technische Universität Dresden, Bergstrasse 66, 01062 Dresden, Germany

Contents

1. Materials and Synthesis S1
2. Single-crystal X-ray diffraction measurements S2
3. Thermogravimetric analysis (TGA) S2
4. Fourier Transform Infrared (FTIR) Spectroscopy S2
5. Powder X-ray diffraction (PXRD) measurements S2
6. Variable Temperature Powder X-ray Diffraction (VT-PXRD) S3
7. *In situ* CO_2 and CH_4 loaded Powder Diffraction S3
8. Low-pressure gas adsorption measurements S4
9. Pore volume calculation based on single crystal data S4
10. High-pressure CO_2 and CH_4 adsorption measurements S4
11. Computational study S5
12. Supporting Figures and Tables S7
13. References S30

1. Materials and Synthesis

All chemicals except (*E*)-3-(pyridin-4-yldiazenyl) benzoic acid (**HL**), 4-aminopyridine, 3-aminobenzoic acid and $NiCl_2{\cdot}6H_2O$ were obtained commercially and used as received without further purification. Synthesis of **HL** was accomplished by a modification of a previously reported procedure.[1]

(*E*)-3-(pyridin-4-yldiazenyl) benzoic acid (HL):

3-Nitrosobenzoic acid: To a solution of 3-aminobenzoic acid (1.0 g, 7.3 mmol) in dichloromethane (12mL), an aqueous solution of Oxone® (8.97 g in 45 mL, 14.6 mmol), was added and the suspension vigorously stirred at room temperature for 1 hour. 3-nitrosobenzoic acid precipitated as a pale solid was isolated by filtration, washed with water, and dried in an 80 °C oven (1.1 g, yield = quant.). ^{1}H NMR (400 MHz, DMSO) δ 8.41 (s, 2H), 8.19 (s, 1H), 7.89 (s, 1H).

HL: Sodium hydroxide (3.00 g, 75 mmol) was dissolved in 100 mL of water to form a 3% NaOH solution. 3-aminopyridine (0.53g, 5.6 mmol) and 3-nitrosobenzoic acid (0.45g, 3.0 mmol) were added and the mixture was heated to reflux overnight. The resulting bright orange solution was then cooled to room temperature, forming a bright orange needle precipitate. Then dissolve the orange precipitate in 30mL water and adjust the pH = 1 with 1M HCl. The solid was filtered, washed with water and dried in a vacuum to give the product (0.5g, 2.2mmol, 73%) as a light orange solid. ^{1}H NMR (400 MHz, DMSO) δ 13.38 (s, 1H), 8.86 (s, 2H), 8.43 (s, 1H), 8.21 (s, 2H), 7.82 (s, 3H). ^{13}C NMR (101 MHz, DMSO) δ 166.95, 156.86, 152.02, 133.51, 130.68, 128.37, 123.16, 116.48.

X-dia-6-Ni-α, $[Ni_2(H_2O)L_4]_n$:

Synthesis: A mixture of $NiCl_2{\cdot}6H_2O$ (0.03 mmol, 7.2 mg), **HL** (0.06 mmol, 13.6 mg), DMF (3mL) and MeOH (1.5 mL) was added to a 10.5 mL glass vial. The vial was capped tightly and placed in an oven at 60 °C for 96h, which was then cooled to room temperature. After rinsing several times with fresh DMF, orange single crystals were obtained. Yield, 60%. IR: ν_{max} (cm^{-1}) = 3066, 2924, 2830, 1666, 1467, 1382, 1268, 1212, 1145, 1060, 833, 748.

X-dia-6-Ni-β, $[Ni_2(H_2O)L_4]_n$:

Synthesis: The as-synthesized open framework (**X-dia-6-Ni-α**) was exchanged with fresh MeOH by using a soxhlet extractor for 2 days and then heated to 60 °C under vacuum for 2h to yield **X-dia-6-Ni-β.** IR: ν_{max} (cm^{-1}) = 3170, 1609, 1545, 1458, 1364, 1229, 1159, 1101, 1025, 931, 832, 785.

X-dia-6-Ni-γ_{EB}, $\{[Ni_2(H_2O)L_4]{\cdot}xEB\}_n$:

Synthesis: The as-synthesized open framework (**X-dia-6-Ni-α**) was exchanged with fresh MeOH and then replace the MeOH with ethylbenzene for 3 days to yield **X-dia-6-Ni-γ_{EB}**.

X-dia-6-Ni-γ_{MX}, $\{[Ni_2(H_2O)L_4]{\cdot}1MX\}_n$:

Synthesis: The as-synthesized open framework (**X-dia-6-Ni-α**) was exchanged with fresh MeOH and then replace the MeOH with m-xylene for 3 days to yield **X-dia-6-Ni-γ_{MX}**.

X-dia-6-Ni-γ_{OX}, $\{[Ni_2(H_2O)L_4]{\cdot}xOX\}_n$:

Synthesis: The as-synthesized open framework (**X-dia-6-Ni-α**) was exchanged with fresh MeOH and then replace the MeOH with o-xylene for 3 days to yield **X-dia-6-Ni-γ_{OX}**.

X-dia-6-Ni-γ_{PX}, {[$Ni_2(H_2O)L_4$]·3PX}$_n$:

Synthesis: The as-synthesized open framework (**X-dia-6-Ni-α**) was exchanged with fresh MeOH and then replace the MeOH with p-xylene for 3 days to yield **X-dia-6-Ni-γ_{PX}**.

2. Single-crystal X-ray diffraction measurements

Single-crystal reflection data were collected on a Bruker Quest diffractometer equipped with a CMOS detector and IμS microfocus X-ray source (Cu K_α, λ = 1.54178 Å; Mo K_α, λ = 0.71073 Å). Indexing was performed using APEX3[2] (Difference Vectors method). Absorption correction was performed by the multi-scan method implemented in SADABS.[3] Space group was determined using XPREP implemented in APEX3.[2] Structural solution and refinement against F^2 were carried out using the SHELXL non-linear least squares implemented in Olex2 v1.2.10.[4,5] All non-hydrogen framework atoms were refined with anisotropic parameters, while H atoms were placed in calculated positions and refined using a riding model. Some disordered atoms have been refined isotropically. Most of the guests in structures could not be determined because of the big voids of pores. All the crystals were measured under liquid N_2 flow at a temperature of 100K or 120K to avoid the phase transformation caused by guest molecules escaping into the air. Crystallographic data and structural refinement information are listed in Table S2. The structure of porous phases **X-dia-6-Ni-α**, **γ_{EB}**, **γ_{PX}** were solved and refined in the *Fddd* space group; **X-dia-6-Ni-γ_{MX}** in the *F222* space group. The structure of the low porosity phase **X-dia-1-Ni-β**, **γ_{OX}** was solved and refined in the *Fdd2* space group. Crystallographic data for the structures reported in this paper have been deposited with the Cambridge Crystallographic Data Centre as supplementary publication No. CCDC 2225285-2225290.

3. Thermogravimetric analysis (TGA)

Thermogravimetric analyses (TGA) were performed under N_2 using a TA Instruments Q50 system. Samples were loaded into aluminium sample pans and heated at 10 K min^{-1} from room temperature to 500 °C.

4. Fourier Transform Infrared (FTIR) Spectroscopy

Spectra were obtained by using a FTIR spectrometer (Agilent technologies, Cary 630) in the range of 4000-650 cm^{-1}.

5. Powder X-ray diffraction (PXRD) measurements

Powder X-ray diffraction patterns were recorded on a PANalytical X'Pert MPD Pro (Cu Ka, λ = 1.5418 Å) with a 1D X'Celerator strip detector. Experiments were conducted in continuous scanning mode

with the goniometer in the theta-theta orientation. Incident beam optics included the Fixed Divergences slit with anti-scatter slit PreFIX module, with a 1/8°divergence slit and a 1/4° anti-scatter slit, as well as a 10 mm fixed incident beam mask and a Soller slit (0.04 rad). Divergent beam optics included a P7.5 anti-scatter slit, a Soller slit (0.04 rad), and a Ni β filter. The data were collected in the range of 2θ = 3 – 40°. Raw data were then evaluated using the X'Pert HighScore Plus™ software V 4.1 (PANalytical, The Netherlands).

6. Variable Temperature Powder X-ray Diffraction (VT-PXRD)

Diffractograms at different temperatures were recorded using a PANalytical X'Pert Pro-MPD diffractometer equipped with a PIXcel3D detector operating in scanning line detector mode with an active length of 4 utilizing 255 channels. The Anton Paar TTK 450 stage and the Anton Paar TCU 110 Temperature Control Unit were used to record the variable temperature diffractograms. The diffractometer is outfitted with an Empyrean Cu LFF (long fine focus) HR (9430 033 7300x) tube operated at 40 kV and 40 mA and CuKα radiation (λ_α = 1.54056 Å) was used for diffraction experiments. The data was collected by continuous scanning mode with the goniometer in the theta-theta orientation. Incident beam optics included the Fixed Divergences slit, with a 1/4° divergence slit and a Soller slit (0.04 rad). Divergent beam optics included a P7.5 anti-scatter slit, a Soller slit (0.04 rad), and a Ni-β filter. In a typical experiment, 20 mg of sample was ground into a fine powder and loaded on a zero-background sample holder made for Anton Paar TTK 450 chamber. The data were collected from 4 - 40° (2θ) with a step size of 0.0167113° and a scan time of 50 seconds per step. Crude data were analyzed using the X'Pert HighScore Plus™ software V 4.1 (PANalytical, The Netherlands). The sample was heated up to 433 K.

7. *In situ* CO_2 and CH_4 loaded Powder Diffraction

Low pressure CO_2 *in situ* PXRD. *In situ* PXRD patterns on **X-dia-6-Ni** in parallel to CO_2 physisorption at 195 K were measured using home-built dedicated instrumentation, based on Empyrean-2 powder X-ray diffractometer (ω–2θ goniometer, alpha1 system) using a customized setup based on ARS DE-102 closed cycle helium cryostat (T = 30–300 ± 0.1 K) and adsorption cell, built of 1.33′′ CF-flange and Beryllium dome. The cell was connected to the low-pressure port of the BELSORP-max (Microtrac MRB) volumetric adsorption instrument. The TTL-trigger was used for establishing the communication between BELSORP-max and Data Collector software and ensure the measurement of adsorption isotherm and PXRD patterns in automated mode. The diffraction experiments were performed using ω–2θ scans in transmission geometry in the range of 2θ = 3–40°. Parallel beam optics (W/Si mirror, hybrid 2 x Ge (220) monochromator, 4 mm mask, primary divergence and secondary antiscatter slits with ¼° opening) was used for the data collection. Pixcel-3D detector in 1D scanning mode (255 active channels) was used for recording of the scattered intensities. A physisorption of CO_2 at temperature 195 K was measured on 60 mg of **X-dia-6-Ni** sample, mounted in the X-ray beam, and PXRD patterns were recorded after equilibration (0.1% of pressure change within 300 s) at selected points of the isotherm. Adsorption and desorption isotherm, measured *in situ* and corresponding PXRD patterns are given in the Figure 3.

High pressure CH_4 *in situ* PXRD. Experiments were carried out on a PANalytical X'Pert PRO

instrument with Debye-Scherrer geometry. Intensity data were recorded using an X'Celerator detector, and 2θ scans in the range of 3−40° were performed with a step size of 0.016° at the scan speed of 0.00843 (°/s) 250s/step. During the experiment the powdered sample was exposed to Cu Kα radiation (λ = 1.5418 Å). The activated sample **X-dia-6-Ni-β** was sealed within a glass capillary (environmental gas cell) and evacuated ex situ. The capillary was then progressively loaded with methane (the pressures selected correspond to events observed in the gas sorption isotherm collected at the same temperature) and its variable pressure PXRD patterns were measured at a constant temperature of 298 K.

8. Low-pressure gas adsorption measurements

For gas sorption experiments, high-purity gases were used as received from BOC Gases Ireland: He (99.999%), N_2 (99.9992%), CO_2 (99.995%), CH_4 (99.9995%). Low pressure (0-1 bar) CO_2 and N_2 sorption isotherms were measured using Micromeritics 3flex instrument. Methanol (MeOH) exchanged **X-dia-6-Ni** was degassed under high vacuum at 60 °C for 10 h on Micromeritics Smart VacPrep instrument. The activated sample (100 mg) were transferred to 3Flex and evacuated at room temperature for 2 hours before the measurements. The temperature at 77 K and 195 K were maintained using a 4L Dewar filled with liquid nitrogen and a dry ice-acetone mixture respectively. Bath temperature of 273 K was precisely controlled with a Julabo ME (v.2) recirculating control system containing a mixture of ethylene glycol and water.

9. Pore volume calculation based on single crystal data

The calculation of pore volume (V_{pore}) of different phases followed the equation below:

$$V_{pore} = \frac{Voids \times \frac{V_{crystal}}{Z} \times N_A}{F_W} \quad \text{Eq. 1}$$

Where V_{pore} is expressed in cubic centimetres per gram of crystal, *Voids* is the guest accessible volume, $V_{crystal}$ is the crystal volume of each phase, *Z* is the number of asymmetric units of crystal in each phase, N_A is the Avogadro constant, *Fw* is the formula weight of crystal in each phase.

Indicative single point pore volumes were determined experimentally using the Microactive software suite under the assumption of approximate validity of the Gurvich rule[6,7] wherein the structural transformations follow a state approaching saturation of the preceding phase during 195 K CO_2 adsorption experiments as described in Eq. 2, where ν_{pore} is the pore volume, $n^{\text{Ads}}_{\text{CO2}}$ is the quantity of gaseous CO_2 adsorbed as determined at STP, and $\rho^{\text{Liq}}_{\text{CO2}}$ is the density of liquid CO_2.

$$V_{pore} = \frac{n^{Ads}_{CO_2}}{\rho^{Liq}_{CO_2}} \quad \text{Eq. 2}$$

The determined volumes are presented in Table S4 and show reasonable agreement with the assignment of phases based on SCXRD-determined solvent-accessible volumes.

10. High-pressure CO_2 and CH_4 adsorption measurements

High-pressure CO_2 and CH_4 sorption experiments were performed using a Hiden Isochema XEMIS microbalance. Temperatures were maintained at 273 K, 285K and 298 K using a Grant LT

Ecocool 150 temperature controller. Activated samples were further outgassed under a secondary vacuum for 3 hours *in situ* before isotherm experiments were conducted. High-pressure cycling experiments were carried out using pressure ramps between 5 and 35 bar or 5 and 65 bar, with 45-minute equilibration stages in each cycle. Excess adsorption and desorption profiles were obtained after applying a buoyancy correction using the crystallographically determined density of each compound. Excess uptakes were further converted to absolute uptakes where relevant by applying Equation 3.

$$N_{Abs} = N_{Exc} + \rho . V_{pore} \quad \text{Eq. 3}$$

Where ρ is the density of the gas at the given experimental pressure and temperature (obtained from the experimental output), and V_{pore} is the pore volume of the adsorbent.

The volumetric capacity for **X-dia-6-Ni** was calculated based on the density of the open phase (0.85 g/cm^3).

11. Computational study

Le Bail refinement of Powder X-ray Diffraction: Regarding the minor shift of the peak positions at low angles (2θ = 4°- 10°) in the *in situ* PXRD patterns during the CO_2 loading process at 195 K from P/P_0 = 0.06 to 0.90, the CO_2-loaded phase of **X-dia-6-Ni-γ_{CO2}** at P/P_0 = 0.06 was assumed to possess similar structure with the CO_2-fully-loaded phase **X-dia-6-Ni-γ_{MX}**. Thus, Le Bail refinement[8] against PXRD data of the **X-dia-6-Ni-γ_{CO2}** phase was performed on the basis of the lattice parameter for **X-dia-6-Ni-γ_{MX}** to acquire accurate lattice parameters, which resulted in cell unit parameters a = 27.12 Å, b = 32.73 Å, c = 35.83Å, α = γ = β = 90°(R_p = 8.69%, R_{wp} = 12.47%). The structure model was constructed according to the structure of **X-dia-6-Ni-γ_{MX}** and further optimized with the GFN1-xTB [9,10] method within fixed lattice constants using the CP2K code[11]. The Le Bail refinement fit of PXRD data result is presented in Figure S18.

Unit cell determination of CO_2-loaded phase: Unit cell determinations for the CO_2 loaded phase were carried out using the X-Cell program embedded in Materials Studio. Lattice parameters and space group were determined from a set of 14 reflections. An orthorhombic unit cell with lattice constants of a = 36.46 Å, b = 32.58 Å, c = 27.34 Å, α = β = γ = 90°, V = 32476.28 Å^3 was indexed. The structure model was constructed according to the structure of **X-dia-6-Ni-γ_{MX}** and optimized with the GFN1-xTB[10] method within fixed lattice constants using the CP2K code.[11] The grand canonical Monte Carlo (GCMC) simulation was performed at 195 K and 100 kPa by RASPA code[12] to obtain the gas-filled structure of **X-dia-6-Ni**. Periodic boundary conditions were applied in three directions. The simulations started with a cycle of 100,000-cycle equilibration steps and were followed by 100,000 production steps. The framework was described with an all-atoms model, the charge of atoms in the framework was calculated by charge-equilibration method and Universal forcefield (UFF)[13] was used in this study. For the guest molecules, CO_2 were modeled with the Transferable Potentials for Phase Equilibria (TraPPE) force field[14]. The framework atoms are supplied in Supplementary CIF-file (ja4c03555_si_002.cif)

Periodic Density Functional Theory (DFT) calculations were performed using the projected augmented wave (PAW) formalism[15] as implemented in the Vienna Ab Initio Simulation Package (VASP 5.4.4),[16,17] on primitive unit cell models of **X-dia-6-Ni-β** and **X-dia-6-Ni-γ**, respectively, employing the BEEF-vdW exchange-correlation functional.[18] First, the structures are optimized in their primitive experimental unit cells in the Γ-point (using the experimental cell parameters) using

the conjugate gradient algorithm, with force and electronic convergence criteria of 0.02 eV/Å and 10^{-5} eV, a Gaussian smearing of 0.05 eV, an energy cut-off of 500 eV, and two unpaired electrons per Ni.

Canonical Monte Carlo (CMC) simulations[19] are performed in Materials Studio[20] to confirm the main binding site locations for CH_4 at 298 K on 1x1x2 supercells of the DFT-optimized primitive cells for **X-dia-6-Ni-β** and **X-dia-6-Ni-γ_{MX}**. The atomic point charges of the frameworks and methane are determined via the charge equilibration (Qeq) method. The point charges used for methane are shown in Figure S25, while the point charges [e] for the framework atoms are supplied in Supplementary CIF-files (**X-dia-6-Ni-β** ja4c03555_si_003.cif and **X-dia-6-Ni-γ_{MX}** ja4c03555_si_004.cif).

For the CMC simulations, the framework's atoms are kept fixed in their DFT-optimized positions, and a fixed loading of one sorbate molecule (per supercell) is chosen. In the canonical ensemble, the Metropolis sampling method considered different moves, such as translation (corresponds to translation of the center-of-mass of selected adsorbate molecule), rotation (rotating the selected adsorbate molecule), regrowth (removing a selected adsorbate molecule from the system and reintroducing it at a random position with random orientation), and conformer (collecting multiple adsorbate conformations), with relative probabilities of 1, 1, 0.1 and 1, respectively. Furthermore, a cut-off distance of 10 Å is applied and the potentials are truncated using a cubic spline with a spline width of 1 Å. The Lennard-Jones (LJ) pair coefficients are taken from the Universal Force Field (UFF)[20] parameters from the Material Studio forcefield library. The Ewald summation[21] method was used to calculate long - range electrostatic interactions.

The CMC simulations included 2×10^6 loading steps, followed by 2×10^6 equilibration steps, and finally, 2×10^6 production steps to ensure reasonable ensemble averages. The output of the CMC simulations was visualized as adsorbate density fields, encompassing the mass-middle points of all successful adsorbate MC moves (see Table S7). We also visualized an isosurface of constant density (isovalue= 6.159×10^{-5} g/cm^3), and coloured it according to the CH_4 interaction energy (see Table S7) for **X-dia-6-Ni-γ_{MX}** and **X-dia-6-Ni-β**.

Grand Canonical Monte Carlo (GCMC) simulations are performed using force field parameters as in the above CMC section, to determine adsorption isotherms for pressures between 0 to 80 bar. Each GCMC simulation included 5×10^6 equilibration steps, followed by 5×10^6 production steps to ensure reasonable ensemble averages. Figure S26 shows the higher CH_4 uptake in the more open **X-dia-6-Ni-γ_{MX}** phase. The transformation from **X-dia-6-Ni-β** to **X-dia-6-Ni-γ_{MX}** and thus the switching mechanism during adsorption happens around 35 bar at 298 K (compare Figure S26 and Figure 4a). At lower pressures (0 to 35 bar), the maximum uptake in the experimental isotherm (see Figure 4a) closely matches the maximal uptake in the simulated isotherm for **X-dia-6-Ni-β**. At higher pressures (~35 to 80 bar), the experimental isotherm falls a bit below the simulated CH_4 adsorption isotherm for **X-dia-6-Ni-γ_{MX}**.

12. Supporting Figures and Tables

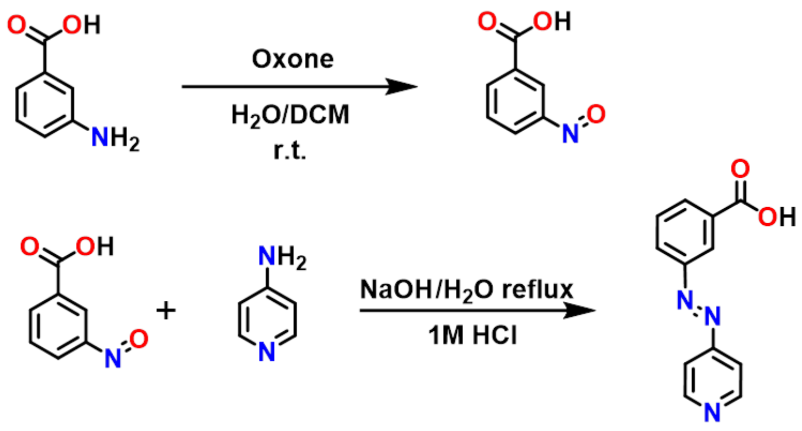

Figure S1. Synthesis of (*E*)-3-(pyridin-4-yldiazenyl) benzoic acid (**HL**).

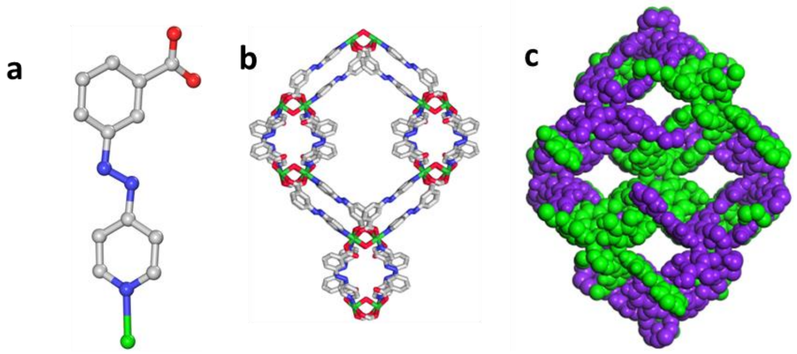

Figure S2. (a) Ligand in **X-dia-6-Ni**. (b) Two layered ligands of adamantanoid cage formed by **L**$^-$ and Ni^{2+}. (c) 2-fold interpenetrated **dia** nets (two different colors) form quadrangular channels along the *b*-axis.

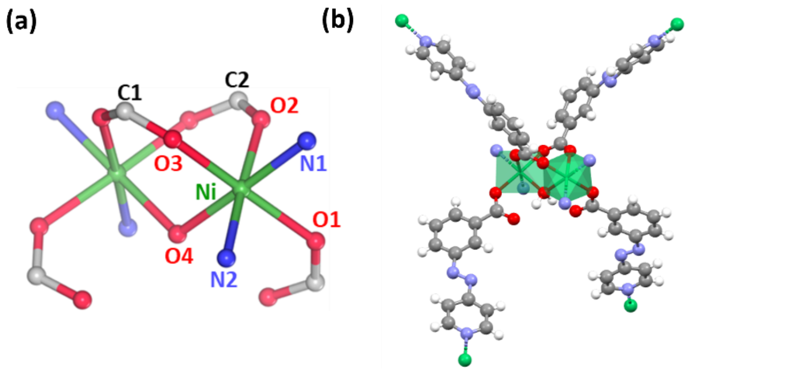

Figure S3. (a) Molecular building block (MBB). (b) Coordination environment in **X-dia-6-Ni**.

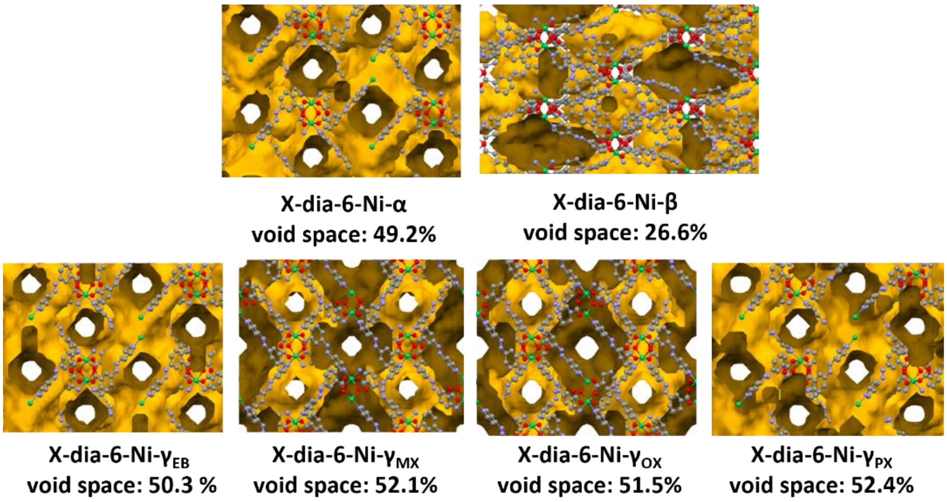

Figure S4. View along the 1D channel pores of **X-dia-6-Ni-α**, **X-dia-6-Ni-β**, **X-dia-6-Ni-γ_{EB}**, **X-dia-6-Ni-γ_{MX}**, **X-dia-6-Ni-γ_{OX}**, **X-dia-6-Ni-γ_{PX}** with various voids.

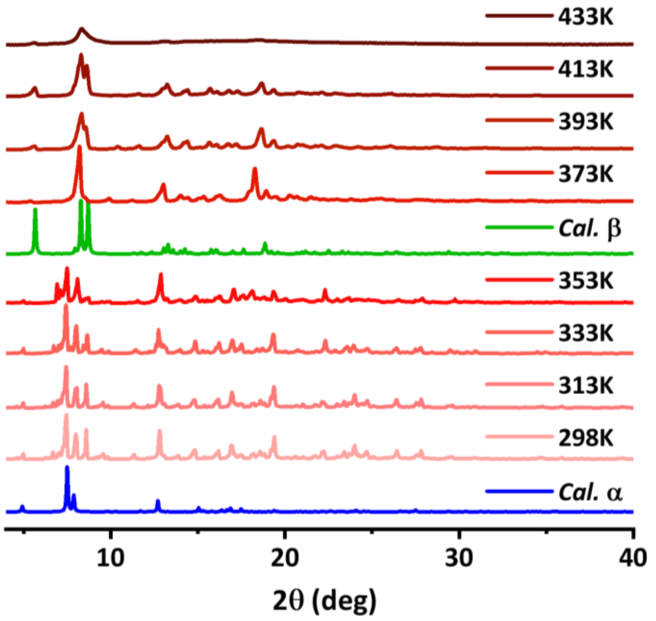

Figure S5. VT-PXRD patterns of as-synthesized **X-dia-6-Ni-α** heated from 298 K to 433 K under nitrogen flow.

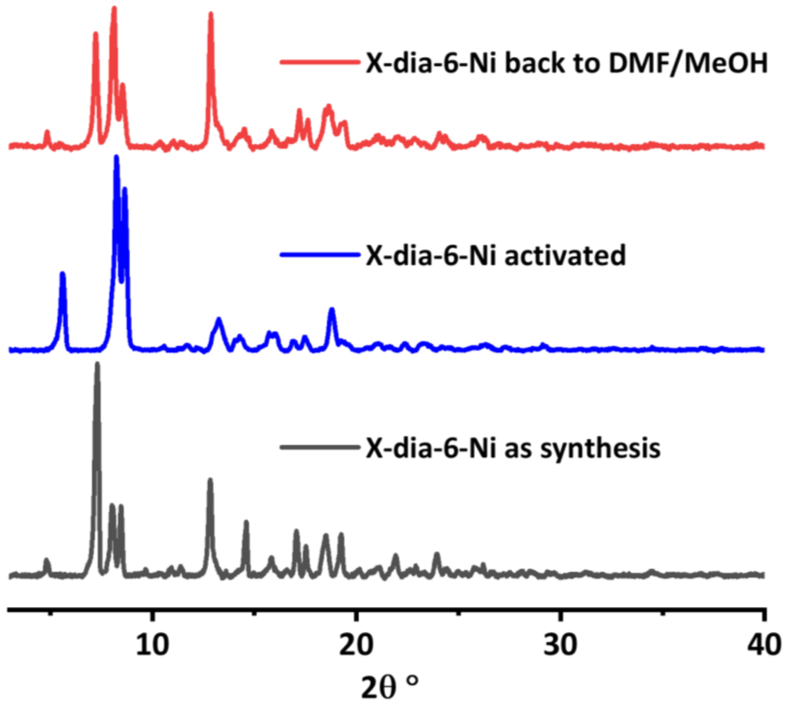

Figure S6. PXRD patterns of **X-dia-6-Ni-α** (as-synthesized), **X-dia-6-Ni-β** (activated), and **X-dia-6-Ni-α** (soaking **β** in DMF/MeOH at 60 °C for 1 day).

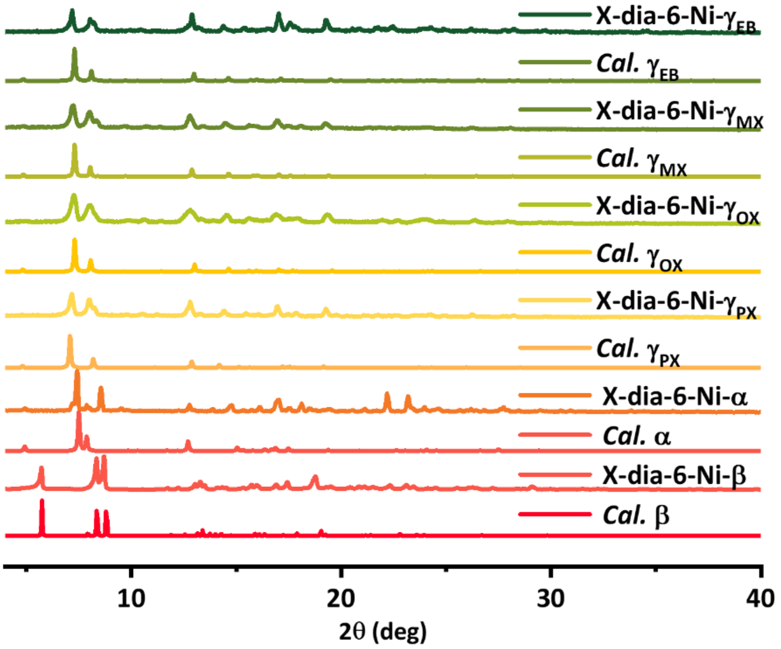

Figure S7. PXRD patterns of **X-dia-6-Ni-β** and **X-dia-6-Ni-α** and activated **β** phase soaked in different C8 hydrocarbon guests which resulted in four distinct phases **X-dia-6-Ni-γ_{EB}**, **X-dia-6-Ni-γ_{MX}**, **X-dia-6-Ni-γ_{OX}**, and **X-dia-6-Ni-γ_{PX}**.

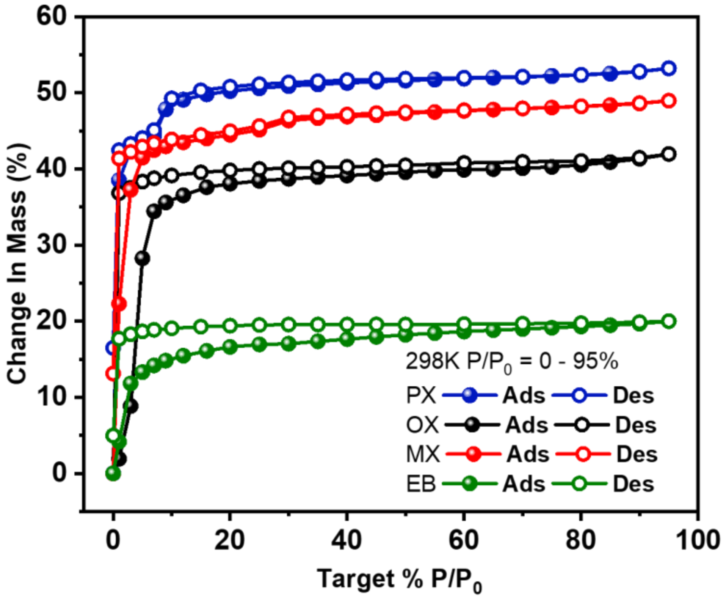

Figure S8. Isotherms of PX, MX, OX and EB collected at 298 K from P/P_0 = 0 – 95% relative pressure.

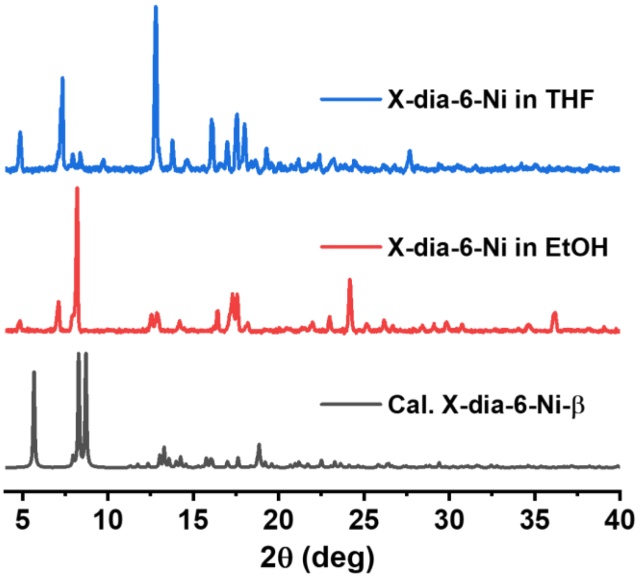

Figure S9. PXRD patterns of **X-dia-6-Ni** soaked in THF (blue), EtOH (red) and calculated PXRD pattern from SCXRD data of **X-dia-6-Ni-β**.

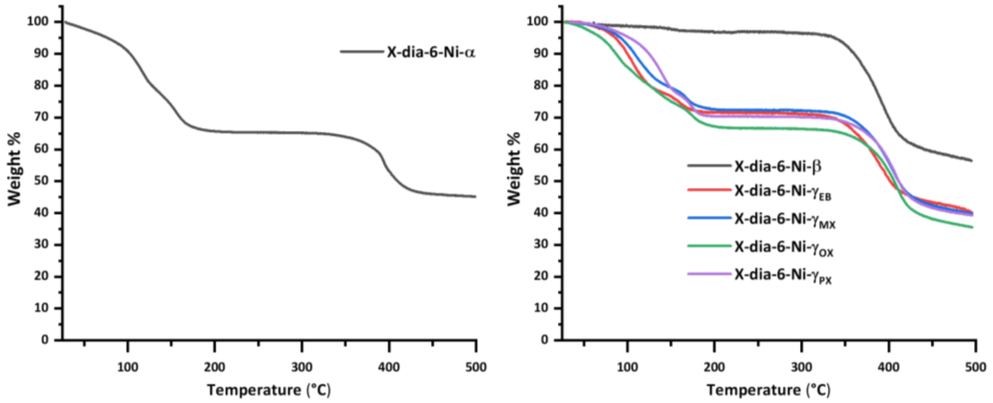

Figure S10. Thermogravimetric analysis of **X-dia-6-Ni-α**, **X-dia-6-Ni-β**, **X-dia-6-Ni-γ$_{EB}$**, **X-dia-6-Ni-γ$_{MX}$**, **X-dia-6-Ni-γ$_{OX}$**, and **X-dia-6-Ni-γ$_{PX}$**.

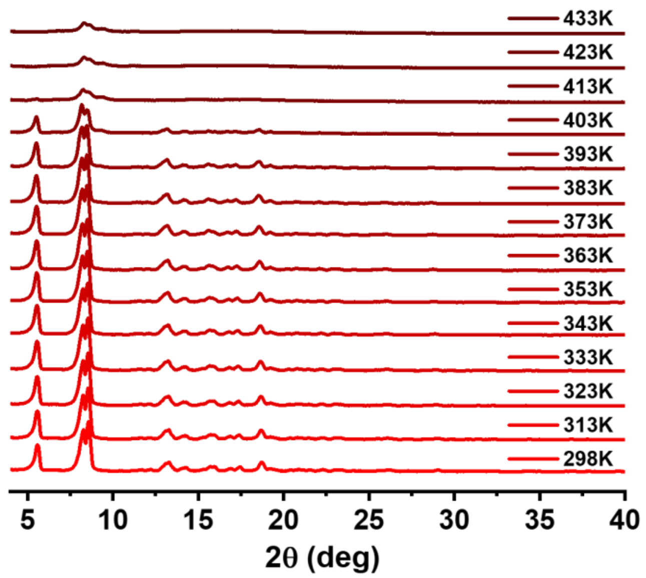

Figure S11. VT-PXRD patterns of activated **X-dia-6-Ni-β** heated from 298 K to 433 K under nitrogen flow.

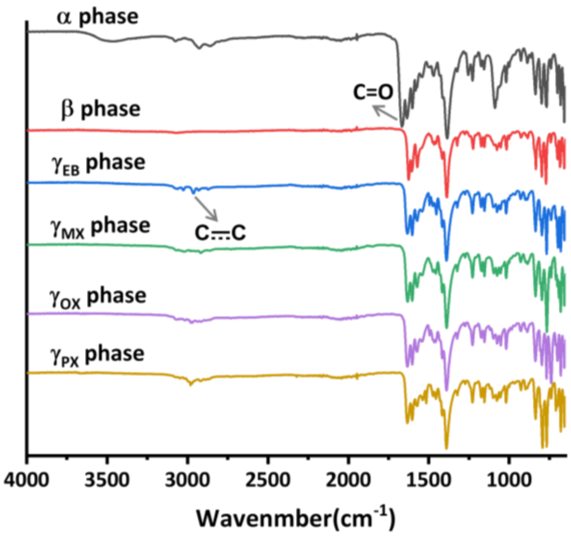

Figure S12. Fourier Transform Infrared (FTIR) spectra of **X-dia-6-Ni-α**, **X-dia-6-Ni-β**, **X-dia-6-Ni-γ_{EB}**, **X-dia-6-Ni-γ_{MX}**, **X-dia-6-Ni-γ_{OX}**, and **X-dia-6-Ni-γ_{PX}**.

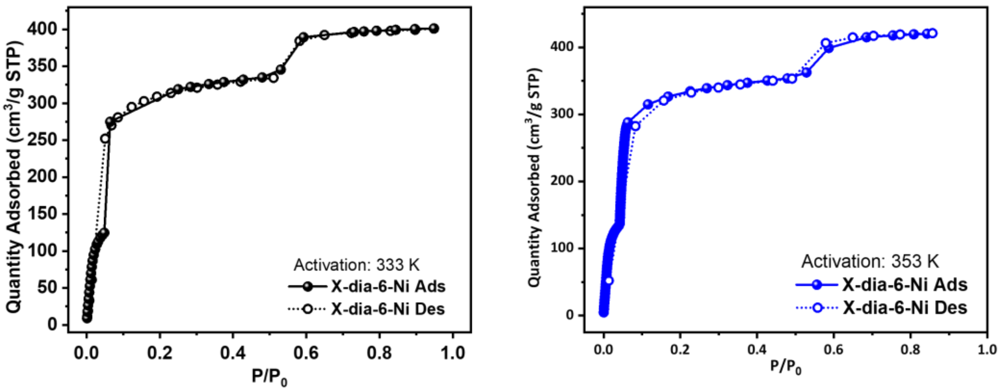

Figure S13. CO_2 Isotherms collected at 195 K with sample of **X-dia-6-Ni** activated at 333 K (black) and 353 K (blue), respectively.

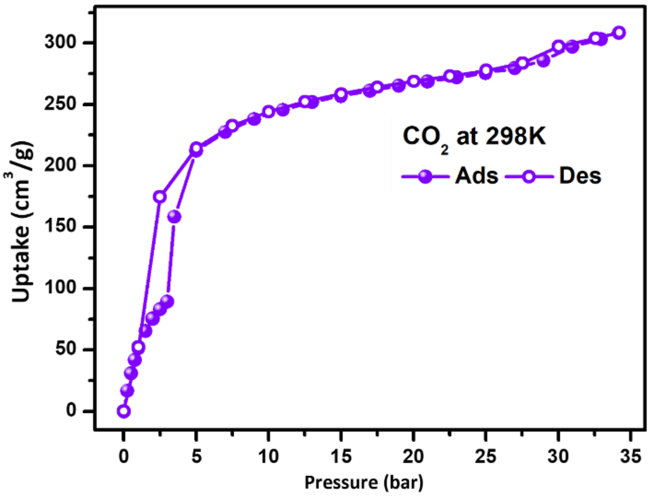

Figure S14. High-pressure sorption isotherm of CO_2 from 0-35 bar at 298 K for **X-dia-6-Ni**.

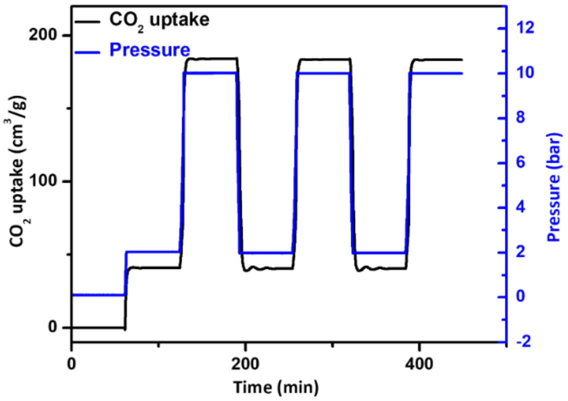

Figure S15. High-pressure cycling experiment of CO_2 for **X-dia-6-Ni** from 2-10 bar at 298 K for 3 cycles.

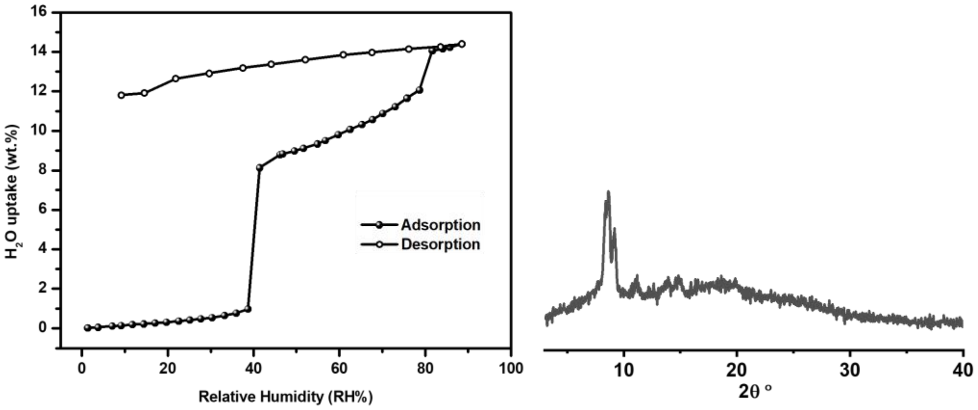

Figure S16. (Right) Water sorption of **X-dia-6-Ni** at 298 K from 0-100% RH. (Left) PXRD pattern of **X-dia-6-Ni** after water sorption test.

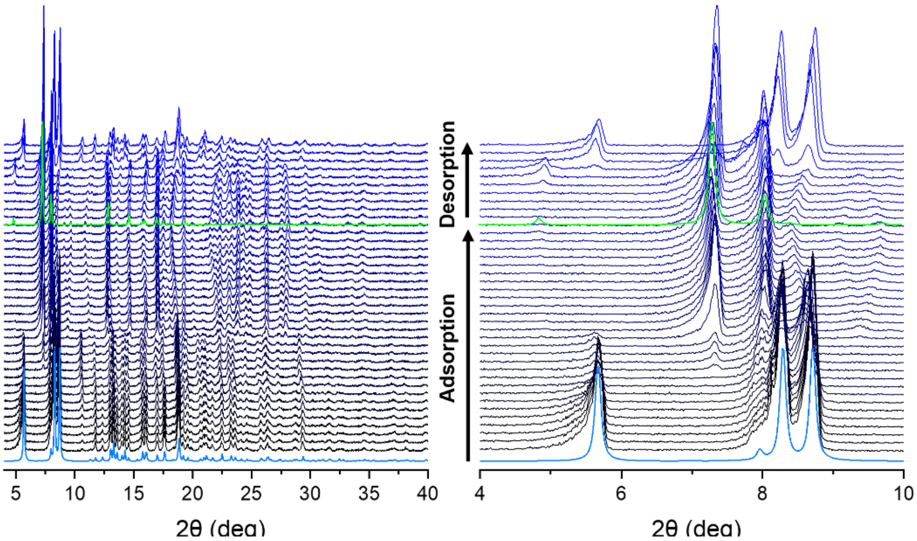

Figure S17. (Left) *In situ* variable pressure PXTD patterns of **X-dia-6-Ni** collected at different CO_2 loading pressures, 195 K (Adsorption P/P_0 = 0-1, desorption P/P_0 = 1-0). (Right) Magnified PXRD patterns from 4 to 10° 2θ values for **X-dia-6-Ni** and comparison with calculated PXRD pattern from single crystal structure of **X-dia-6-Ni-β** (light blue line) and **X-dia-6-Ni-γ_{MX}** (green line).

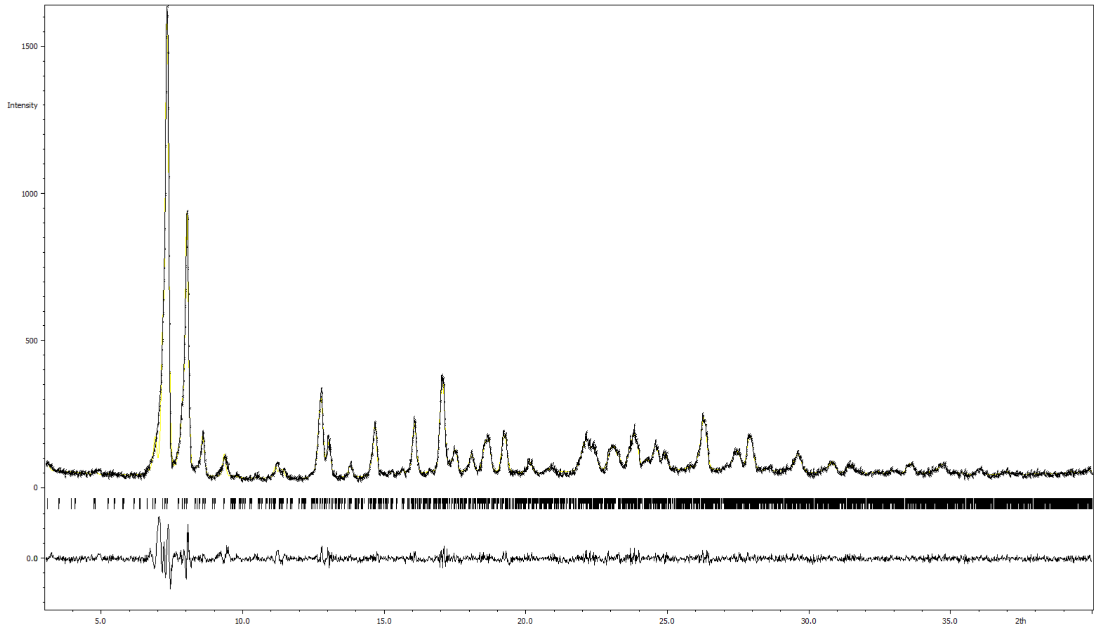

Figure S18. Pawley refinement fit of PXRD data for **X-dia-6-Ni-γ$_{CO2}$**.

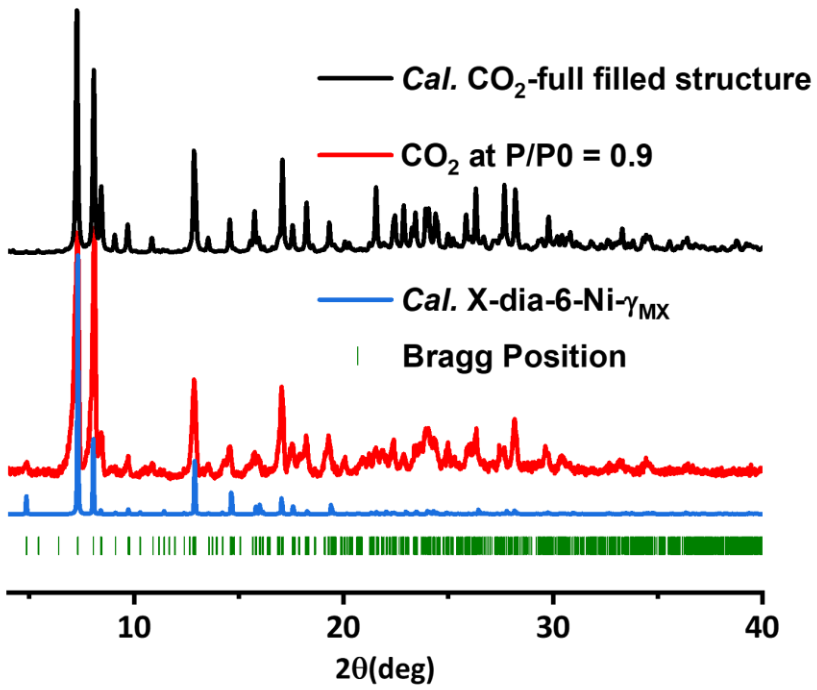

Figure S19. Calculated PXRD pattern from simulated structure of CO_2-full loaded structure; *In situ* PXRD pattern of **X-dia-6-Ni** at CO_2 saturated pressure (P/P_0 = 0.9); calculated PXRD pattern from single crystal data of **X-dia-6-Ni-γ$_{MX}$**; Bragg position from single crystal data of **X-dia-6-Ni-γ$_{MX}$**.

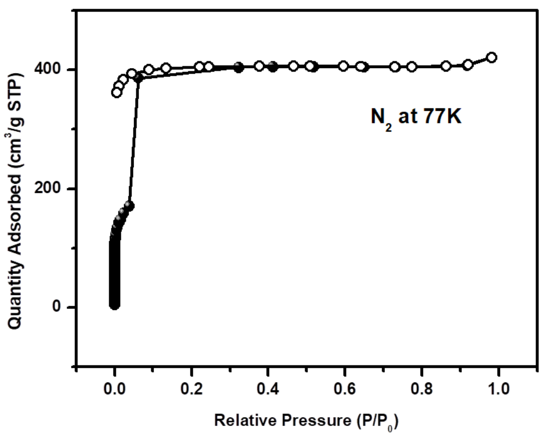

Figure S20. N_2 sorption isotherm at 77 K for **X-dia-6-Ni.**

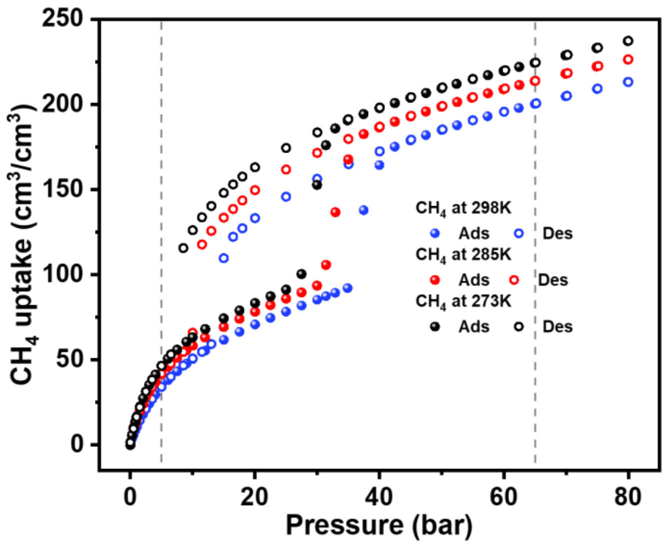

Figure S21. CH_4 sorption isotherm at 273 K, 285 K and 298 K, 0-80 bar for **X-dia-6-Ni.**

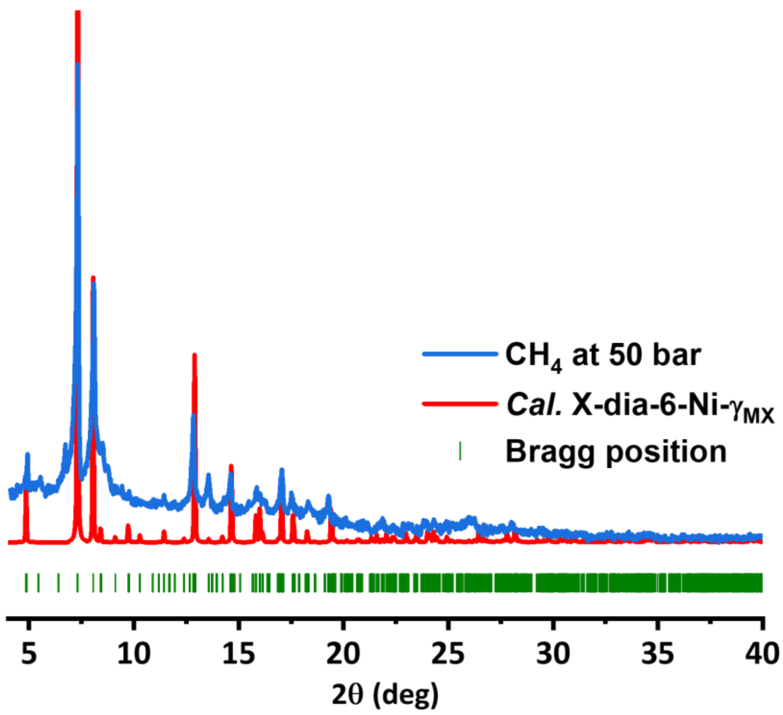

Figure S22. *In situ* PXRD pattern of **X-dia-6-Ni** at CH_4 pressure (P = 50 bar); calculated PXRD pattern from single crystal data of **X-dia-6-Ni-γ$_{MX}$**; Bragg position.

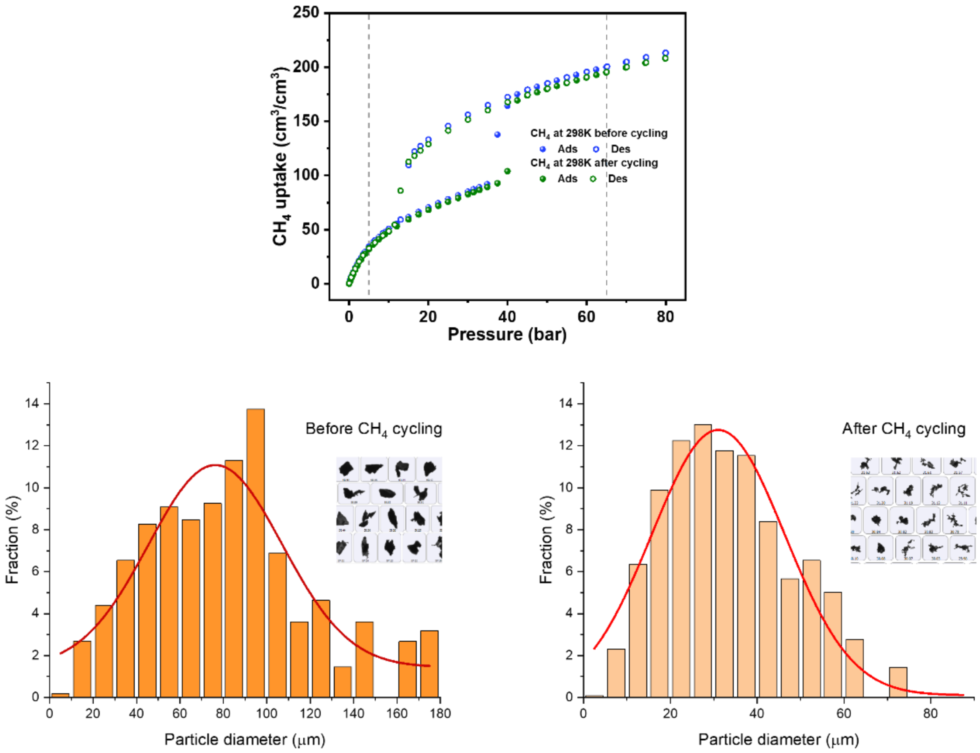

Figure S23. Top: Isotherms of sample before and after 33 CH_4 cycles collected at 298 K. Particle size distribution of **X-dia-6-Ni** before (bottom left) and after (bottom right) 33 cycles CH_4 sorption.

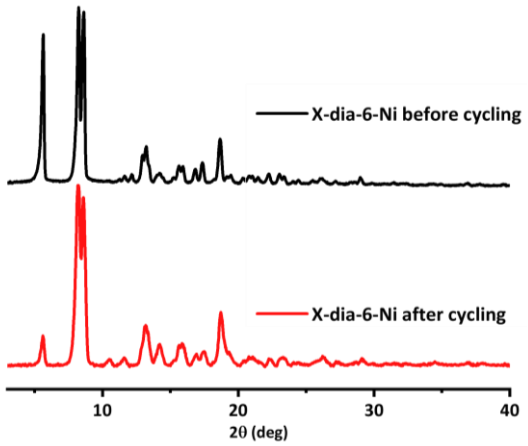

Figure S24. PXRD patterns of **X-dia-6-Ni-β** before and after CH_4 30 cycles cycling experiments.

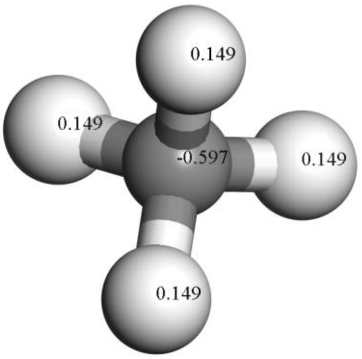

Figure S25. Point charges employed for $\mathbf{CH_4}$ in CMC and GCMC simulations.

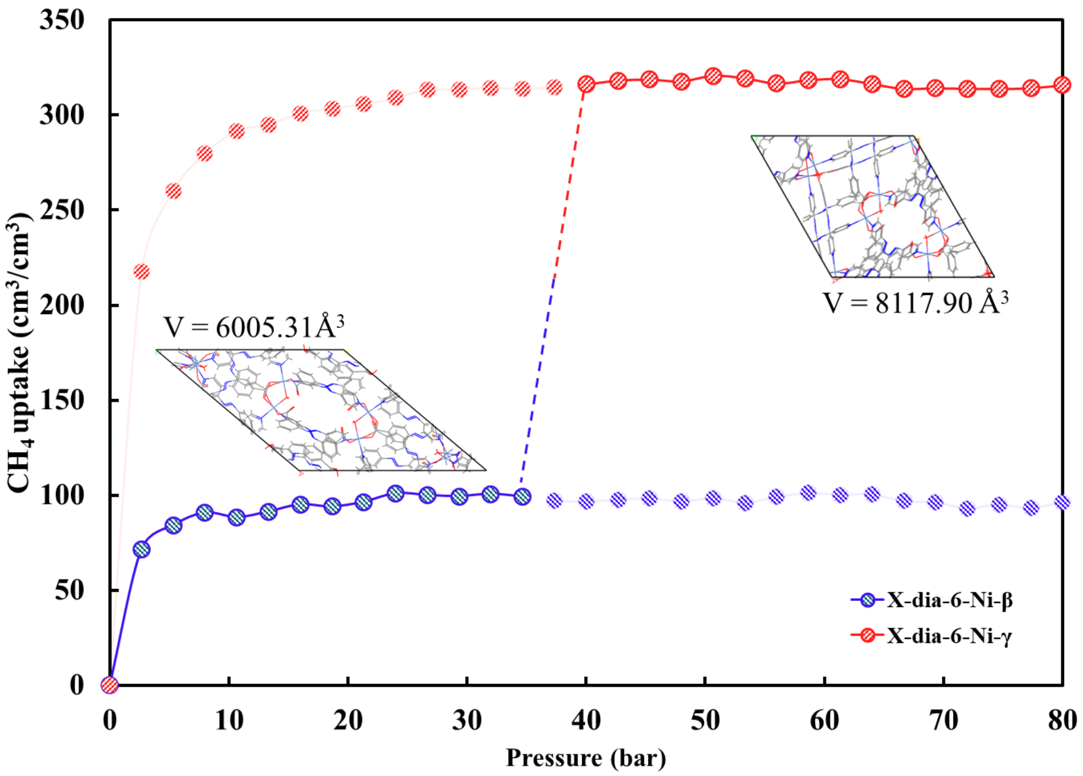

Figure S26. Methane sorption isotherms from GCMC simulations for **X-dia-6-Ni-β** and **X-dia-6-Ni-γ** at 298 K. The dotted line represents the experimentally observed transition or 'switch' between closed and open phase, enabling a higher CH_4 uptake.

Table S1. Crystallographic data and structure refinement summary for **X-dia-6-Ni**.

Compounds	X-dia-6-Ni-α *(High porosity)*	X-dia-6-Ni-β *(Low porosity)*	X-dia-6-Ni-γ_{EB} *(Higher porosity)*
CCDC number	2225285	2225286	2225287
Empirical formula	$C_{24}H_{17}N_6NiO_{4.5}$	$C_{48}H_{34}N_{12}Ni_2O_9$	$C_{24}H_{17}N_6NiO_{4.5}$
Formula weight	520.14	1040.29	520.14
Temperature/K	120	100	100
Crystal system	orthorhombic	orthorhombic	orthorhombic
Space group	*Fddd*	*Fdd2*	*Fddd*
a/Å	27.6693(7)	22.4414(13)	27.5487(5)
b/Å	29.8486(6)	23.9828(13)	33.0208(6)
c/Å	38.2589(8)	44.632(2)	35.6158(7)
α/°	90	90	90
β/°	90	90	90
γ/°	90	90	90
Volume/Å^3	31597.6(12)	24021(2)	32399.0(10)
Z	32	16	32
ρcalcg/cm^3	0.875	1.151	0.853
μ/mm^{-1}	0.519	1.244	0.922
F(000)	8544	8544	8544
Radiation	MoKα (λ = 0.71073)	CuKα (λ = 1.54178)	CuKα (λ = 1.54178)
2Θ range for data collection/°	5.854 to 50.696	5.746 to 133.19	4.858 to 127.37
Index ranges	-33 ⩽ h ⩽ 14, -33 ⩽ k ⩽ 35, -46 ⩽ l ⩽ 38	-22 ⩽ h ⩽ 26, -24 ⩽ k ⩽ 28, -52 ⩽ l ⩽ 46	-32 ⩽ h ⩽ 31, -38 ⩽ k ⩽ 38, -41 ⩽ l ⩽ 40
Reflections collected	20621	46373	59517
Independent reflections	7221 [R_{int} = 0.0482, R_{sigma} = 0.0622]	9757 [R_{int} = 0.1099, R_{sigma} = 0.0952]	6615 [R_{int} = 0.0466, R_{sigma} = 0.0265]
Data/restraints/parameters	7221/0/321	9757/111/643	6615/26/321
Goodness-of-fit on F^2	1.081	0.994	1.067
Final R indexes [I>=2σ (I)]	$R1^a$ = 0.0654, $wR2^b$ = 0.2048	$R1^a$ = 0.0889, $wR2^b$ = 0.2352	$R1^a$ = 0.0874, $wR2^b$ = 0.2514
Final R indexes [all data]	$R1^a$ = 0.0800, $wR2^b$ = 0.2135	$R1^a$ = 0.1218, $wR2^b$ = 0.2725	$R1^a$ = 0.0914, $wR2^b$ = 0.2555
Largest diff. peak/hole / e Å^{-3}	0.60/-0.57	0.81/-0.46	1.19/-0.75

$^aR_1=\Sigma||F_o|-|F_c||/\Sigma|F_o|$. $^bwR_2= [\Sigma w(|F_o|^2-|F_c|^2)|/\Sigma|w(Fo^2)^2|^{1/2}$

Compounds	X-dia-6-Ni-γ_{MX} *(Higher porosity)*	X-dia-6-Ni-γ_{OX} *(Higher porosity)*	X-dia-6-Ni-γ_{PX} *(Higher porosity)*
CCDC number	2225290	2225289	2225288
Empirical formula	$C_{56}H_{44}N_{12}Ni_2O_9$	$C_{48}H_{34}N_{12}Ni_2O_9$	$C_{36}H_{32}N_6NiO_{4.5}$
Formula weight	1046.45	1040.29	679.38
Temperature/K	100	100	100
Crystal system	orthorhombic	orthorhombic	orthorhombic
Space group	*F222*	*Fdd2*	*Fddd*
a/Å	27.6082(11)	28.1657(5)	26.9570(6)
b/Å	32.4685(15)	32.9293(6)	34.5881(7)
c/Å	36.2242(17)	35.1682(6)	35.9786(8)
α/°	90	90	90
β/°	90	90	90
γ/°	90	90	90
Volume/Å^3	32471(2)	32617.7(10)	33546.1(13)
Z	16	16	32
ρcalcg/cm^3	0.938	0.847	1.076
μ/mm^{-1}	0.956	0.916	0.994
F(000)	9472	8544	11328
Radiation	CuKα (λ = 1.54178)	CuKα (λ = 1.54178)	CuKα (λ = 1.54178)
2Θ range for data collection/°	8.408 to 149.89	4.832 to 144.86	8.464 to 149.128
Index ranges	-34 ⩽ h ⩽ 34, -39 ⩽ k ⩽ 40, -45 ⩽ l ⩽ 40	-33 ⩽ h ⩽ 34, -40 ⩽ k ⩽ 39, -43 ⩽ l ⩽ 43	-33 ⩽ h ⩽ 33, -38 ⩽ k ⩽ 43, -44 ⩽ l ⩽ 43
Reflections collected	90196	85807	92447
Independent reflections	16625 [R_{int} = 0.0608, R_{sigma} = 0.0441]	15141 [R_{int} = 0.0653, R_{sigma} = 0.0400]	8530 [R_{int} = 0.0662, R_{sigma} = 0.0333]
Data/restraints/parameters	16625/63/714	15141/115/642	8530/36/431
Goodness-of-fit on F^2	1.075	1.058	1.063
Final R indexes [I>=2σ (I)]	R1^a = 0.0852, wR2^b = 0.2423	R1^a = 0.0614, wR2^b = 0.1764	R1^a = 0.0822, wR2^b = 0.2226
Final R indexes [all data]	R1^a = 0.0930, wR2^b = 0.2540	R1^a = 0.0701, wR2^b = 0.1869	R1^a = 0.0886, wR2^b = 0.2294
Largest diff. peak/hole / e Å^{-3}	0.71/-0.82	1.23/-0.52	1.80/-1.00

$^aR_1=\Sigma||F_o|-|F_c||/\Sigma|F_o|$. $^bwR_2=|\Sigma w(|F_o|^2-|F_c|^2)|/\Sigma|w(Fo^2)^2|^{1/2}$

Table S2. CSD (Version 5.44, April 2023) search results of reported networks with double walled **dia** topology.

Refcode	Application	Refcode	Application	Refcode	Application
AMUWIP	Rigid structure	HAZPOP	Rigid structure	POMXIA	Rigid structure
BAHCIZ	Catalysis	HOHJAR	Magnetic	QOJXUK	Rigid structure
BIYTEK	Magnetic	IBIYOI	Rigid structure	SATBIA	Rigid structure
CEJFOO	Magnetic	KEYDEZ	Rigid structure	UGOXUJ	Rigid structure
CEJFUU	Magnetic	KUTCUZ	Catalysis	UGOYAQ	Rigid structure
CEJGAB	Magnetic	KUTDAG	Catalysis	UGOYEU	Rigid structure
DIBWUH	Magnetic	LASBIS	Magnetic	UJAFET	Rigid structure
DIBXAO	Magnetic	LITHUR	Rigid structure	UNAHEY	Luminescent and magnetic
DIVFOF	Magnetic	LUPDEH	Rigid structure	VEHXEN	Rigid structure
EKEJOU	Rigid structure	LUWZUZ	Rigid structure	VOCKUT	Rigid structure
EKEKAH	Rigid structure	MOFNED	Photocatalysis	VOCQAF	Rigid structure
FASYUU	Rigid structure	MORFEG	Magnetic	VOCQEJ	Rigid structure
FELRUL	Rigid structure	MOYTUR	Rigid structure	VUJWUT	Rigid structure
GINKOG	Sensing	NERVEL	Rigid structure	WIWQOI	Rigid structure
GUPRIU	Rigid structure	NERVIP	Rigid structure	WIWQUO	Rigid structure
GURXIB	Rigid structure	NERVOV	Rigid structure	WOPWOP	Rigid structure
GURXOH	Rigid structure	NIMWUD	Fluorescent	XAPNOT	Rigid structure
GURXUN	Rigid structure	NUDLEF	Photocatalytic	XASHAA	Rigid structure
HAZNED	Rigid structure	OFEGOX	Rigid structure	XIRKAM	Catalytic
HAZNIH	Rigid structure	OPADOA	Rigid structure	ZATPIW	Flexible
HAZNON	Rigid structure	PAFPAQ	Rigid structure	ZATPOC	Flexible
HAZPAB	Rigid structure	PAQFIY	Photocatalytic	ZEFLAZ	Rigid structure
WURNOO	Rigid structure	KIFBOT	Rigid structure	VOFWEV	Rigid structure
XOVHIC01	Rigid structure	XOXGUN	Rigid structure		

Table S3. CSD (Version 5.44, April 2023) search results of reported networks with $\{Ni_2\}$ MBB $\{Ni_2(COO)_2(OH_2)N_2O_2\}$.

No.	CSD refcode	Dimension (topology or node connectivity)	No.	CSD refcode	Dimension (topology or node connectivity)
1	OFILEY	3D (**ddi**)	60	LICROF	3D (5-connected)
2	OFILIC	3D (**ddi**)	61	LOSRUJ	0D
3	OFILUO	3D (**ddi**)	62	LOSSAQ	0D
4	OFIMAV	3D (**ddi**)	63	LUKNIP	0D
5	OFIMID	3D (**ddi**)	64	MELRUT	3D (8-connected)
6	WIDDOG	0D	65	MOJHUQ	0D
7	ACATEN	0D	66	NEVBOG	0D
8	ACLMEN	0D	67	NEVCAT	0D
9	ADUFEN	0D	68	NEVCIB	0D
10	APRNIM	0D	69	NEVCOH	0D
11	AXARAV	0D	70	NEXLOV	0D
12	AZAYAB	0D	71	OCOZIS	0D
13	BAXLUH	0D	72	OCUXUF	0D
14	BAXLUH01	0D	73	PICBOS	0D
15	BONMAT	3D (8-connected)	74	POJBUO	0D
16	CAENNI	0D	75	POJBUO01	0D
17	CATSOI	0D	76	POJBUO02	0D
18	CETGOY	0D	77	POJCAV	0D
19	CLPMNI	0D	78	POJCAV01	0D
20	CONNEY	0D	79	POQZAZ	3D (6-connected)
21	CPRENI10	0D	80	PUSFIV	0D
22	CUMCIX	3D (6-connected)	81	PUSXOQ	0D
23	CUNHIE	0D	82	QAGZAA	0D
24	DUFSUU	0D	83	QAGZEE	0D
25	ECICUS	2D	84	QAVTEO	1D
26	FAGBAT	0D	85	QOJXUK	3D (double walled **dia**)
27	FAGRIR	0D	86	QORTAU	2D
28	FATLUI	1D	87	RUTPOM	1D
29	FUMQOW	0D	88	SETMEK	0D
30	FUMQOW01	0D	89	SOFZUJ	0D
31	FUMQUC	0D	90	UCAGAJ	0D
32	FUMQUC01	0D	91	UHESIJ	0D
33	FURGOP	0D	92	UHESOP	0D
34	GEDJAC	0D	93	UJAFET	3D (double walled **dia**)
35	GISYEM	0D	94	UKITAJ	0D
36	GISYIQ	0D	95	UKOVEY	0D
37	GISYOW	0D	96	UKOVIC	0D
38	GODPEV	3D (6-connected)	97	UTIQEV	2D
39	HAKPER	0D	98	VANNOO	0D
40	HUQQIT	0D	99	VAVBAX	2D

41	IBERIU	0D	100	VAVBEB	2D
42	ILEQOF	0D	101	VOCNOQ	3D (6-connected)
43	ILEQUL	1D	102	VOCQEJ	3D (double walled **dia**)
44	INESIE	3D (5-connected)	103	VOFWEV	3D (double walled **dia**)
45	IPULOV	0D	104	VOWCIV	3D (5-connected)
46	IQISUW	0D	105	WAKQOP	0D
47	IWOCUU	2D	106	WEJZEQ	0D
48	IYOBOP	3D (**msw**)	107	WORNIA	0D
49	IYOFEJ	0D	108	WOVDES	3D (6-connected)
50	JITTIS	0D	109	WUZRUE	3D (5-connected)
51	JONRAH	0D	110	XEQSIW	0D
52	JONYAO	0D	111	XOVHIC01	3D (double walled **dia**)
53	JOQCEX	0D	112	XOXGUN	3D (single walled **dia**)
54	KANHIT	0D	113	YOJJUE	0D
55	KANHIT01	0D	114	YURWEO	0D
56	KEXYOD	0D	115	YUTNUZ	2D
57	KIFBOT	3D (8-connected **dia**)	116	ZEGDOI	0D
58	KOPVES	0D	117	ZUGROK	2D
59	LALMUI	0D			

Table S4. Comparative analysis of the structural differences between the three isolated phases (six structures) of **X-dia-6-Ni**.

		**X-dia-6-Ni-α**	**X-dia-6-Ni-β**[a]	**X-dia-6-Ni-γ_{EB}**
Coordination geometry	Bond length and bond angle	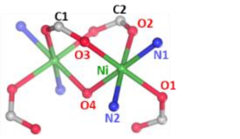	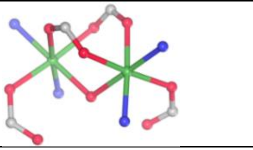	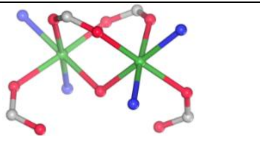
	Ni-O1 (Å)<br>Ni-O2 (Å)<br>Ni-O3 (Å)<br>Ni-O4 (Å)<br>Ni-N1 (Å)<br>Ni-N2 (Å)<br>∠C1-O4-C2 (°)	2.047(3)<br>2.056(3)<br>2.010(3)<br>2.094(2)<br>2.083(4)<br>2.114(4)<br>69.52(9)	1.993(8), 2.039(8)<br>2.066(8), 2.085(8)<br>2.031(8), 2.098(7)<br>2.121(6), 2.042(5)<br>2.119(10), 2.049(10)<br>2.082(5), 2.223(5)<br>55.85(22), 58.66(33)	2.041(2)<br>2.089(2)<br>2.006(3)<br>2.109(2)<br>2.133(3)<br>2.110(3)<br>72.24(9)
Dihedral angles between carboxyl planes connected with the same two Ni^{2+} cations		89.61(46)<br>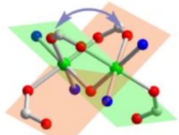	22.55(18)°,<br>34.69(17)°<br>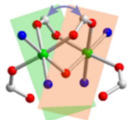	76.11(42)°<br>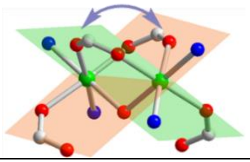
Dihedral angles between the carboxyl plane and benzoate plane of **L**$^-$.<br>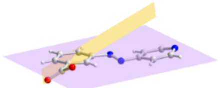		3.10(42) °,<br>13.76(36) °	29.83(63)°,<br>74.34(35)°,<br>8.84(12)°,<br>2.13(75)	7.83(20)°,<br>13.75(21)°
Dihedral angles between the pyridyl plane and benzoate plane of **L**$^-$.<br>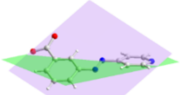		17.71(26)°,<br>43.33(56)°	7.67(47)°,<br>40.63(40)°,<br>23.90(40)°,<br>50.59(71)°	18.29(24)°,<br>33.03(19)°
Edge lengths and angles of channel pores along the *b* axis		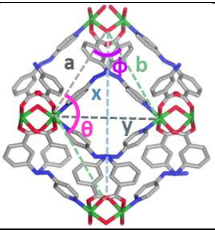	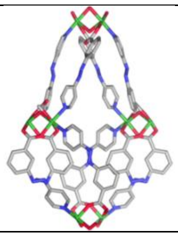	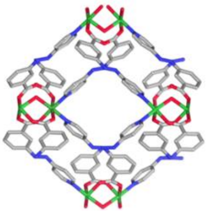
a (Å)<br>b (Å)<br>x (Å)<br>y (Å)<br>ϕ(°)<br>θ(°)		13.071(3)<br>12.945(3)<br>17.073(6)<br>10.660(1)<br>48.38(1)<br>82.03(1)	13.197(8)<br>13.072(8)<br>20.317(13)<br>8.480(3)<br>37.66(2)<br>101.93(2)	13.054(2)<br>12.975(2)<br>15.646(5)<br>10.529(1)<br>47.72(1)<br>73.90(1)
View along *a* axis of **X-dia-6-Ni's** 2-folded interpenetrated **dia** nets structure with two different colors		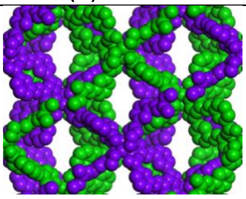	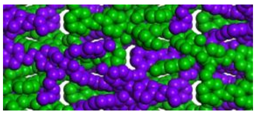	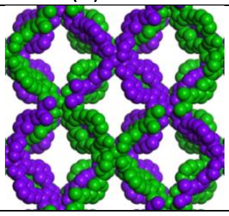

[a] *The multiple values generated from the low symmetry of the structure.*

		X-dia-6-Ni-γ_{MX}[a]	X-dia-6-Ni-γ_{OX}[a]	X-dia-6-Ni-γ_{PX}
Coordination geometry	Bond length and bond angle	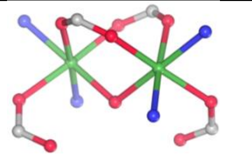	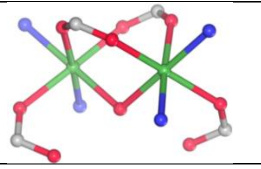	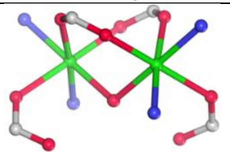
	Ni-O1 (Å)<br>Ni-O2 (Å)<br>Ni-O3 (Å)<br>Ni-O4 (Å)<br>Ni-N1 (Å)<br>Ni-N2 (Å)<br>∠C1-O4-C2 (°)	2.060(3), 2.034(3)<br>2.065(3), 2.061(3)<br>2.031(3), 2.006(3)<br>2.106(3), 2.098(2)<br>2.093(4), 2.120(3)<br>2.111(4), 2.120(3)<br>71.23(10), 71.10(8)	2.051(4), 2.024(4)<br>2.030(5), 2.087(4)<br>1.997(4), 2.017(4)<br>2.138(3), 2.104(3)<br>2.072(5), 2.121(4)<br>2.125(5), 2.079(5)<br>72.27(15), 72.00(13)	2.069(2)<br>2.046(2)<br>2.023(2)<br>2.104(2)<br>2.095(2)<br>2.097(2)<br>73.83(7)
Dihedral angles between carboxyl planes connected with the same two Ni^{2+} cations		76.97(98)°,<br>81.90(46)°<br>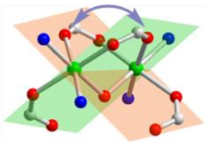	75.68(61)°,<br>77.01(66)°<br>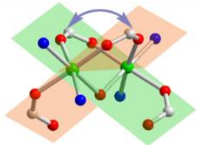	69.78(37)°<br>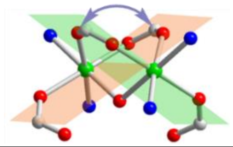
Dihedral angles between the carboxyl plane and benzoate plane of **L**$^-$.<br>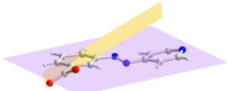		11.52(30)°,<br>5.86(27)°,<br>16.27(28)°,<br>6.82(22) °	5.52(37)°,<br>18.96(27)°,<br>9.82(40)°,<br>4.19(38)°	13.37(41)°,<br>28.44(23)°
Dihedral angles between pyridyl plane and benzoate plane of **L**$^-$.<br>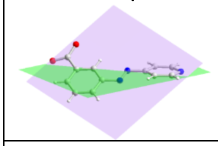		18.67(23) °,<br>33.81(23) °,<br>17.25(28) °,<br>37.10(25) °	10.94(29)°,<br>29.72(22)°,<br>14.01(30)°,<br>41.55(20)	24.20(45) °,<br>28.92(19) °
Edge lengths and angles of channel pores along the *b* axis		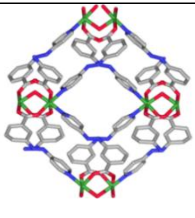	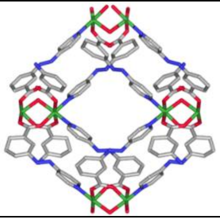	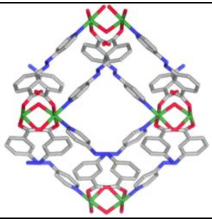
a (Å)<br>b (Å)<br>x (Å)<br>y (Å)<br>ϕ(°)<br>θ(°)		13.023(3)<br>12.989(3)<br>15.960(8)<br>10.545(1)<br>47.83(1)<br>75.70(1)	12.933(4)<br>13.005(4)<br>15.406(9)<br>10.814(1)<br>49.28(1)<br>72.78(1)	13.320(2)<br>13.610(2)<br>16.188(4)<br>10.332(1)<br>45.10(1)<br>73.89(1)
View along *a* axis of **X-dia-6-Ni's** 2-folded interpenetrated **dia** nets structure with two different colors		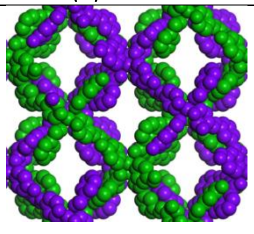	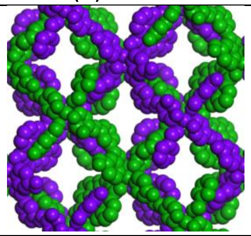	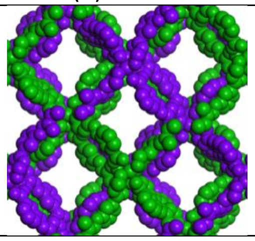

[a] *The multiple values generated from the low symmetry of the structure.*

Table S5. Comparison of experimental pore volume in 195 K CO_2 isotherm and calculated pore volumes of **X-dia-6-Ni-β**, **X-dia-6-Ni-γ_{CO2}** and **X-dia-6-Ni-γ_{MX}** phases.

**Pressure points (P/P_0)**	**Experimental pore volume (cm^3/g)**	**Calculated pore volume (cm^3/g)**
0.042	0.196	0.195 (calculated from **X-dia-6-Ni-β**)
0.479	0.533	0.570 (calculated from simulated structure **X-dia-6-Ni-γ_{CO2}**)
0.843	0.600	0.608 (calculated from **X-dia-6-Ni-γ_{MX}**)

Table S6. Summary of FMOMs with methane storage ability.

**Networks**	**Type of CH_4 sorption isotherms at 298 K or 303 K**	**Topology**[22]	**CH_4 retained at 298 K, 5 bar (cm^3/cm^3)**	**CH_4 uptake at 298 K, 65 bar (cm^3/cm^3)**	**CH_4 working capacity at 298 K, 5-65 bar (cm^3/cm^3)**	**CH_4 gate opening pressure (P_{GO}, bar)**	**N_2 uptake at 77 K (cm^3/g)/P_{GO} (bar)[a]**	**CO_2 uptake at 195 K (cm^3/g)/P_{GO} (bar)[a]**	**Refs**
Co(bpd)	Type F-IV	**snp**	6	203	197	298 K, 16	656.3/3E-3	N/A	10.1038/nature15732
Fe(bpd)	Type F-IV	**snp**	4	196	190	298 K, 24	629.4/2E-3	N/A	10.1038/nature15732
**X-dia-6-Ni**	Type F-II	**dia**	34[b]	200[b]	166[b]	298 K, 40	422/0.037	401/0.047	This work
MIL-53 (Al)-OH	Type F-IV	**sra**	25	217	164	298 K, 15	468.75/N/A	N/A	10.1021/acs.chemmater.8b05332
X-dia-1-$Ni_{0.89}Co_{0.11}$	Type F-IV	**dia**	19 (cm^3/g)	221 (cm^3/g)	202 (cm^3/g)	298 K, 25	no uptake	325/0.05	10.1002/cssc.202300069
X-dia-1-Ni	Type F-IV	**dia**	40	189	149	298 K, 20	N/A	325/0.01	10.1002/anie.201800820
MIL-53 (Al)-$(OH)_2$	Type F-IV	**sra**	20	96	71	298 K, 53	437.5/0.03	N/A	10.1021/acs.chemmater.8b05332
MIL-53 (Fe)	Type F-IV	**sra**	N/A	N/A	N/A	303 K, 10	N/A	N/A	10.1021/ja902740r
$Cu_2(bdc)_2(bpy)$	Type F-IV	**pcu**	45 (cm^3/g)	N/A	N/A	298 K, 9	170/10E-5	N/A	10.1039/B110899A
$[Cu(dhbc)_2(bpy)]$	Type F-IV	**pcu**	20 (cm^3/g)	77 (cm^3/g)	57 (cm^3/g)	298 K, 8	35/N/A (100/N/A)	N/A	10.1002/anie.200390130
$Cu(bpy)_2(BF_4)_2$	Type F-IV	**sql**	0	130	130	303 K, 38	238/0.2	420/0.003	10.1021/jp052515o<br>[d]10.1016/j.micromeso.2012.01.008<br>[e]10.1021/acs.langmuir.6b0255

MIL-53(Al)-NH_2	Type F-IV	**sra**	6	121	115	298 K, 24	400/0.02	N/A	10.1039/C7CC02743E
Co(F-bdp)	Type F-IV	**snp**	14	N/A	N/A	298 K, 5.7	560/3E-4	N/A	10.1021/jacs.6b09155
Co(p-F2-bdp)	Type F-IV	**snp**	3.5	N/A	N/A	298 K, 10	538/2E-3	N/A	10.1021/jacs.6b09155
Co(o-F2-bdp)	Type F-IV	**snp**	3.4	N/A	N/A	298 K, 11	616/1E-3	N/A	10.1021/jacs.6b09155
Co(D4-bdp)	Type F-IV	**snp**	2	N/A	N/A	298 K, 18	616/8E-4	N/A	10.1021/jacs.6b09155
ZIF-7	Type F-IV	**sod**	9	93	84	303 K, 10	20.2/N/A (560/0.05)	N/A (112/0.12)	10.1021/jacs.8b09631 [f]10.1016/j.cej.2021.130117 [g]10.1002/chem.201100958
ZIF-9	Type F-IV	**sod**	6	95	89	303 K, 15	17.9/N/A (20/N/A)	N/A (45/0.25 at 273 K)	10.1021/jacs.8b09631 [h]10.1002/jctb.5947
DUT-49[c]	Type F-II	**fcu**	N/A	N/A	N/A	111 K, 0.45	1850/0.1	N/A	10.1039/C2CC34840C
MIL-53(Al)[c]	Type I	**sra**	60	N/A	N/A	196 K, 1	400/N/A	N/A	10.1021/jp108710h [i]10.1007/s10934-009-9320-5

[a]Values were estimated from reported graphs for uniformity of data.

[b]The volumetric capacity for X-dia-6-Ni was calculated based on the density of the open phase (0.85 g/cm^3).

[c]Low temperature CH_4 sorption.

Note: The extra references of d-i are added for providing the missing values in original references. Topologies of reported materials are obtained in their reported works or Ref.8.

Table S7. The adsorbate density fields from CMC simulations for **CH_4** at 298 K allow the identification of optimal binding sites. At the isosurface of constant density (isovalue = 6.159 x 10^{-5} g/cm^3), the potential CH_4 adsorption energy can be coloured in (kcal/mol, from low (red) to high (blue) adsorption energy).

Structure	CH_4 density fields	CH_4 subsurface color by mapped potential field
**X-dia-6-Ni-β**	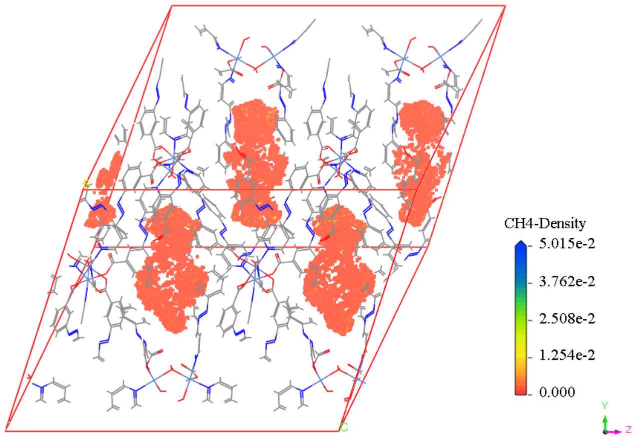	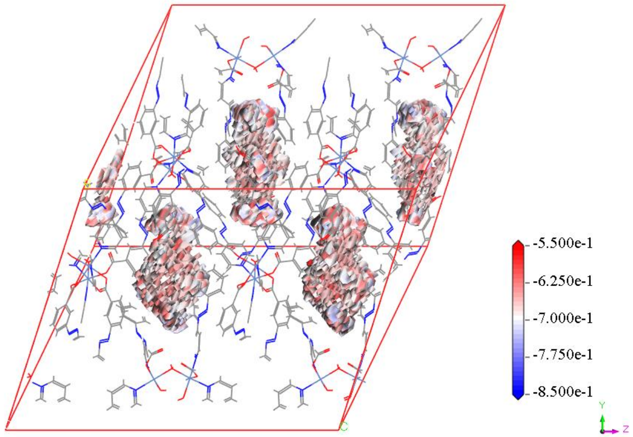
**X-dia-6-Ni-γ**	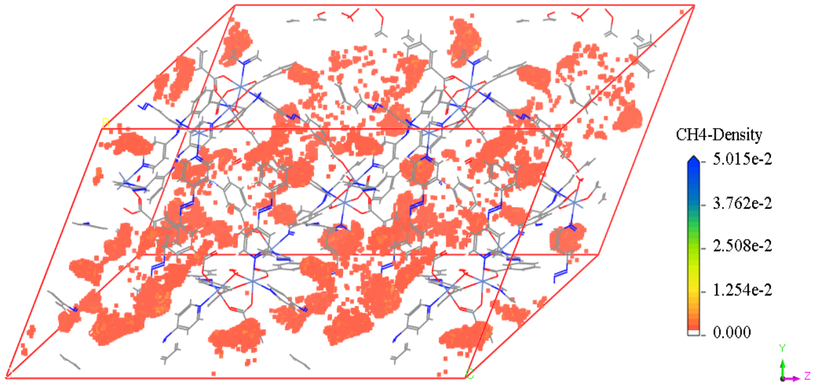	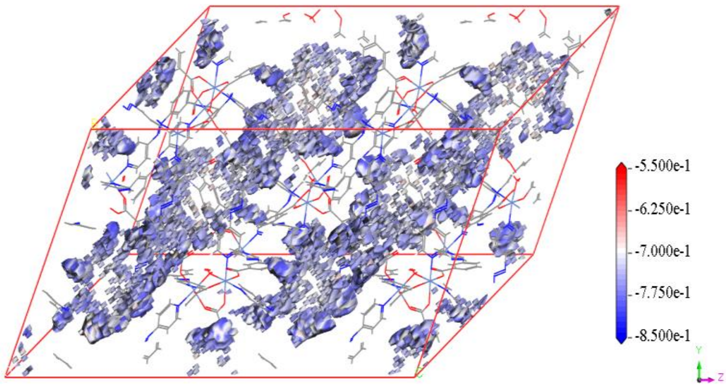

13. References

(1) Oldknow, S.; Martir, D. R.; Pritchard, V. E.; Blitz, M. A.; Fishwick, C. W. G.; Zysman-Colman, E.; Hardie, M. J. Structure-Switching M3L2 Ir(Iii) Coordination Cages with Photo-Isomerising Azo-Aromatic Linkers. *Chem. Sci.* **2018**, *9* (42), 8150–8159. https://doi.org/10.1039/c8sc03499k.

(2) Sheldrick, G. M.; Bruker, A. X. S. Inc., Madison, WI, 2000;(b) GM Sheldrick. *Acta Crystallogr., Sect. A Fundam. Crystallogr* **2015**, *71*, 3–8.

(3) Krause, L.; Herbst-Irmer, R.; Sheldrick, G. M.; Stalke, D. Comparison of Silver and Molybdenum Microfocus X-Ray Sources for Single-Crystal Structure Determination. *J. Appl. Crystallogr.* **2015**, *48* (1), 3–10. https://doi.org/10.1107/S1600576714022985.

(4) Dolomanov, O. V.; Bourhis, L. J.; Gildea, R. J.; Howard, J. A. K.; Puschmann, H. OLEX2: A Complete Structure Solution, Refinement and Analysis Program. *J. Appl. Crystallogr.* **2009**, *42* (2), 339–341. https://doi.org/10.1107/S0021889808042726.

(5) Sheldrick, G. M. Crystal Structure Refinement with SHELXL. *Acta Crystallogr. Sect. C Struct. Chem.* **2015**, *71* (Md), 3–8. https://doi.org/10.1107/S2053229614024218.

(6) Gurvich, L. J. Cited in SJ Gregg, KSW Sing, Adsorption, Surface Area and Porosity, Academic Press, London, P124, 1982. As. *J. Phys. Chem. Soc. Russ* **1915**, *47* (1), 49–56.

(7) Islamoglu, T.; Idrees, K. B.; Son, F. A.; Chen, Z.; Lee, S.-J.; Li, P.; Farha, O. K. Are You Using the Right Probe Molecules for Assessing the Textural Properties of Metal–Organic Frameworks? *J. Mater. Chem. A* **2022**, *10* (1), 157–173. https://doi.org/10.1039/D1TA08021K.

(8) Petříček, V.; Dušek, M.; Palatinus, L. Crystallographic Computing System JANA2006: General Features. *Zeitschrift für Krist. Mater.* **2014**, *229* (5), 345–352.

(9) Grimme, S.; Bannwarth, C.; Shushkov, P. A Robust and Accurate Tight-Binding Quantum Chemical Method for Structures, Vibrational Frequencies, and Noncovalent Interactions of Large Molecular Systems Parametrized for All Spd-Block Elements (Z = 1–86). *J. Chem. Theory Comput.* **2017**, *13* (5), 1989–2009. https://doi.org/10.1021/acs.jctc.7b00118.

(10) Bannwarth, C.; Caldeweyher, E.; Ehlert, S.; Hansen, A.; Pracht, P.; Seibert, J.; Spicher, S.; Grimme, S. Extended Tight-Binding Quantum Chemistry Methods. *WIREs Comput. Mol. Sci.* **2021**, *11* (2), e1493. https://doi.org/https://doi.org/10.1002/wcms.1493.

(11) Kühne, T. D.; Iannuzzi, M.; Del Ben, M.; Rybkin, V. V; Seewald, P.; Stein, F.; Laino, T.; Khaliullin, R. Z.; Schütt, O.; Schiffmann, F. CP2K: An Electronic Structure and Molecular Dynamics Software Package-Quickstep: Efficient and Accurate Electronic Structure Calculations. *J. Chem. Phys.* **2020**, *152* (19).

(12) Dubbeldam, D.; Calero, S.; Ellis, D. E.; Snurr, R. Q. RASPA: Molecular Simulation Software for Adsorption and Diffusion in Flexible Nanoporous Materials. *Mol. Simul.* **2016**, *42* (2), 81–101. https://doi.org/10.1080/08927022.2015.1010082.

(13) Rappé, A. K.; Casewit, C. J.; Colwell, K. S.; Goddard, W. A.; Skiff, W. M. UFF, a Full Periodic Table Force Field for Molecular Mechanics and Molecular Dynamics Simulations. *J. Am. Chem. Soc.* **1992**, *114* (25), 10024–10035. https://doi.org/10.1021/ja00051a040.

(14) Potoff, J. J.; Siepmann, J. I. Vapor–Liquid Equilibria of Mixtures Containing Alkanes, Carbon Dioxide, and Nitrogen. *AIChE J.* **2001**, *47* (7), 1676–1682.

(15) Blöchl, P. E. Projector Augmented-Wave Method. *Phys. Rev. B* **1994**, *50* (24), 17953–17979. https://doi.org/10.1103/PhysRevB.50.17953.

(16) Kresse, G.; Furthmüller, J. Efficient Iterative Schemes for Ab Initio Total-Energy Calculations Using a Plane-Wave Basis Set. *Phys. Rev. B - Condens. Matter Mater. Phys.* **1996**, *54* (16), 11169–11186. https://doi.org/10.1103/PhysRevB.54.11169.

(17) Kresse, G.; Furthmüller, J. Efficiency of Ab-Initio Total Energy Calculations for Metals and Semiconductors Using a Plane-Wave Basis Set. *Comput. Mater. Sci.* **1996**, *6* (1), 15–50. https://doi.org/10.1016/0927-0256(96)00008-0.

(18) Wellendorff, J.; Lundgaard, K. T.; Møgelhøj, A.; Petzold, V.; Landis, D. D.; Nørskov, J. K.; Bligaard, T.; Jacobsen, K. W. Density Functionals for Surface Science: Exchange-Correlation Model Development with Bayesian Error Estimation. *Phys. Rev. B - Condens. Matter Mater. Phys.* **2012**, *85* (23), 235149. https://doi.org/10.1103/PhysRevB.85.235149.

(19) Frenkel, D.; Smit, B. *Understanding Molecular Simulation: From Algorithms to Applications*; Academic Press San Diego, **2002**; Vol. 1.

(20) BIOVIA, Dassault Systèmes, Materials Studio, San Diego: Dassault Systèmes, **2023**.

(21) Wells, B. A.; Chaffee, A. L. Ewald Summation for Molecular Simulations. *J. Chem. Theory Comput.* **2015**, *11* (8), 3684–3695.

(22) Schoedel, A.; Li, M.; Li, D.; O'Keeffe, M.; Yaghi, O. M. Structures of Metal–Organic Frameworks with Rod Secondary Building Units. *Chem. Rev.* **2016**, *116* (19), 12466–12535. https://doi.org/10.1021/acs.chemrev.6b00346.
